# Metabolism and DNA Adduct Formation of Tobacco-Specific *N*-Nitrosamines

**DOI:** 10.3390/ijms23095109

**Published:** 2022-05-04

**Authors:** Yupeng Li, Stephen S. Hecht

**Affiliations:** 1 Masonic Cancer Center, University of Minnesota, Minneapolis, MN 55455, USA; hecht002@umn.edu; 2 Department of Medicinal Chemistry, College of Pharmacy, University of Minnesota, Minneapolis, MN 55455, USA

**Keywords:** metabolism, DNA adducts, tobacco-specific *N*-nitrosamines, NNK, NNN

## Abstract

The tobacco-specific *N*-nitrosamines 4-(*N*-nitrosomethylamino)-1-(3-pyridyl)-1-butanone (NNK) and *N*′-nitrosonornicotine (NNN) always occur together and exclusively in tobacco products or in environments contaminated by tobacco smoke. They have been classified as “carcinogenic to humans” by the International Agency for Research on Cancer. In 1998, we published a review of the biochemistry, biology and carcinogenicity of tobacco-specific nitrosamines. Over the past 20 years, considerable progress has been made in our understanding of the mechanisms of metabolism and DNA adduct formation by these two important carcinogens, along with progress on their carcinogenicity and mutagenicity. In this review, we aim to provide an update on the carcinogenicity and mechanisms of the metabolism and DNA interactions of NNK and NNN.

## 1. Introduction

**Scheme 1:**
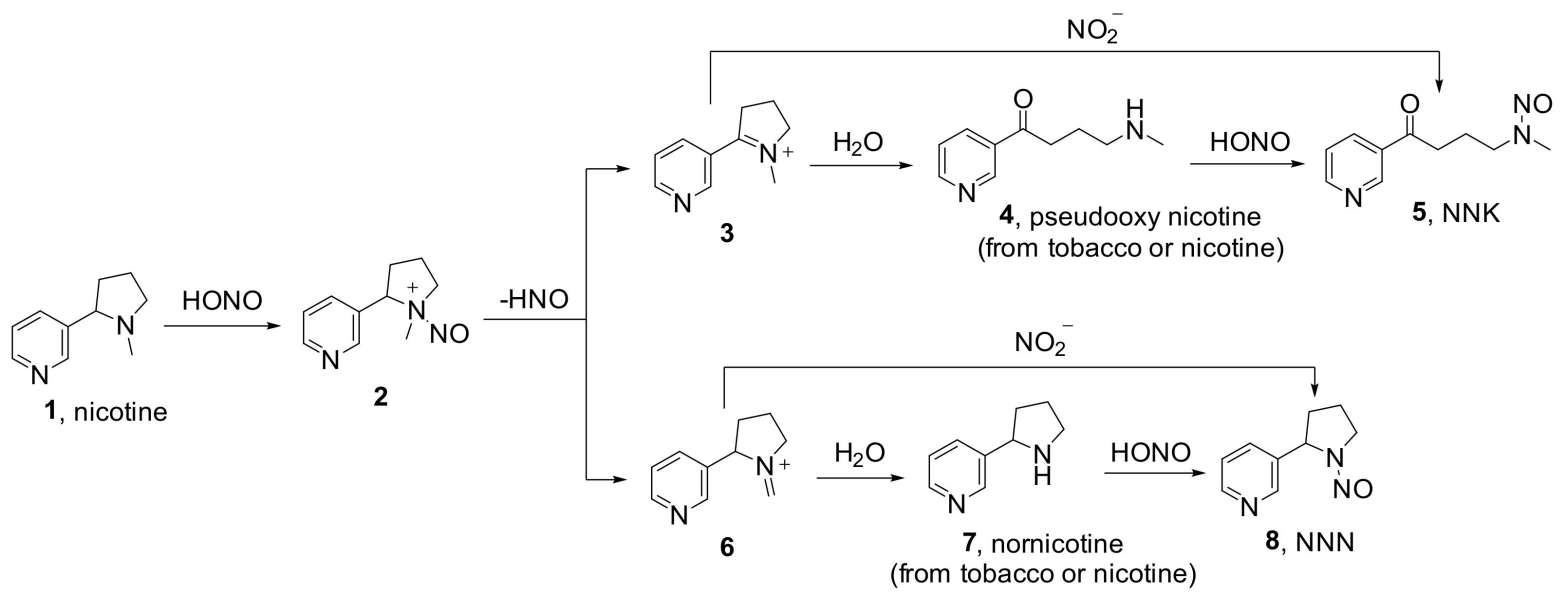
Mechanisms of formation of tobacco-specific *N*-nitrosamines.

Tobacco use remains the leading preventable cause of cancer death [1]. Among over 80 carcinogens identified in tobacco and tobacco smoke [2], the tobacco-specific *N*-nitrosamines (TSNAs) 4-(*N*-nitrosomethylamino)-1-(3-pyridyl)-1-butanone **5** (NNK, Scheme 1) and *N*′-nitrosonornicotine **8** (NNN) are particularly noteworthy carcinogens that have been linked to lung, oral cavity, and esophageal cancers in tobacco users. While the levels of commonly occurring nitrosamines, such as *N*-nitrosodimethylamine (NDMA) and *N*-nitrosopyrrolidine (NPYR), are generally fairly low in tobacco products and tobacco smoke, NNN and NNK are consistently present in remarkably higher abundance.

The formation of NNK and NNN mainly result from the nitrosation of their precursor amines in tobacco pseudooxy nicotine **4** and nornicotine **7** [3,4]. They can also be formed by nitrosation of nicotine **1**, the overwhelmingly abundant tobacco alkaloid, which is not carcinogenic but is highly addictive [5].

Since the comprehensive 1998 review of the biochemistry, biology and carcinogenicity of NNK and NNN [6], significant progress has been made in our understanding the of the metabolism and DNA interactions of these nitrosamines, while several reviews have discussed aspects of the occurrence, metabolism, biomarker and relevant cancer etiology studies of these two carcinogens [7,8,9,10,11], but no comprehensive review of NNK and NNN metabolism and DNA adduct formation has been published. In this paper, we aim to provide an update on the metabolism and DNA interactions of NNK and NNN. Recent carcinogenicity and mutagenicity data are also included.

## 2. Human Exposure to Carcinogenic Tobacco-Specific *N*-Nitrosamines

The major source of human exposure to total *N*-nitrosamines in the U.S. is the consumption of tobacco products, which is estimated to be maximally 25,000 ± 4950 ng per day, in sharp contrast to other sources from food (1900 ± 380 ng/day), alcohol (1000 ± 200 ng/day) and drinking water (120 ± 24 ng/day). The tobacco-specific *N*-nitrosamines NNN, NNK, *N*′-nitrosoanatabine (NAT) and *N*′-nitrosoanabasine (NAB) predominated among the total *N*-nitrosamines reported in tobacco and tobacco smoke [12].

Since the first identification of NNN in unburned tobacco and tobacco smoke in the 1970s [13,14], multiple studies have evaluated the levels of TSNAs in various tobacco products [2]. In one recent study of 50 U.S. domestic cigarette products, the levels of these nitrosamines in tobacco filler were (mean ± S.D.): NNN, 1900.7 ± 359; NNK, 522.8 ± 157.8; NAB, 72.6 ± 12.4; and NAT, 1386.5 ± 261 ng/g tobacco. In mainstream smoke, they were: NNN, 85 ± 31; NNK, 55 ± 20; NAB, 11.7 ± 3.5; and NAT, 80 ± 28 ng/cigarette. The levels in the mainstream smoke of a 2R4F reference cigarette were: NNN, 133.1 ± 12.5; NNK, 115.6 ± 8.7; NAB, 11.7 ± 3.5; and NAT 119 ± 8.5 ng/cigarette [15]. Other recent studies have compared the levels of TSNAs in smokeless tobacco products consumed in different parts of the world [16,17]. The levels of NNN plus NNK were higher in snus products sold in the U.S. than in those sold in northern Europe [17], while the highest levels of these carcinogens in smokeless tobacco products were found in South-East Asia [9]; for example, the levels of total TSNAs in 22 zarda brands and 4 gul brands ranged 6.3–114 µg/g and 35–56 µg/g tobacco powder, respectively [16]. The high levels of these carcinogens, particularly NNN, in the smokeless tobacco products consumed in South-East Asia are especially notable, since (*S*)-NNN, the enantiomer that predominates in smokeless tobacco, is a powerful oral-cavity carcinogen in rats. Thus, NNN likely plays an important role in oral cancer induction in smokeless tobacco users. NNK, on the other hand, is a potent inducer of lung tumors in all the animal species tested, independently of the route of administration at low doses. Collectively, the extensive data on the NNN and NNK levels in tobacco products, coupled with the demonstrated carcinogenicity of these products in humans, led to the IARC classification of NNN and NNK, which always occur together in tobacco products, as “carcinogenic to humans” [18].

## 3. Metabolism and DNA Adduct Formation of NNK and Its Metabolite, NNAL

### 3.1. Occurrence and Carcinogenicity

NNK is considered to mainly result from the nitrosation of pseudooxy nicotine **4** (Scheme 1), which is present in tobacco in both free and matrix-bound forms [3]. It also results from reactions with nicotine during the curing process. The reaction of nicotine and nitrite in vitro leads to the formation of NNK, with a favorable pH range of 3.4–4.5 [5,19].

**Scheme 2:**
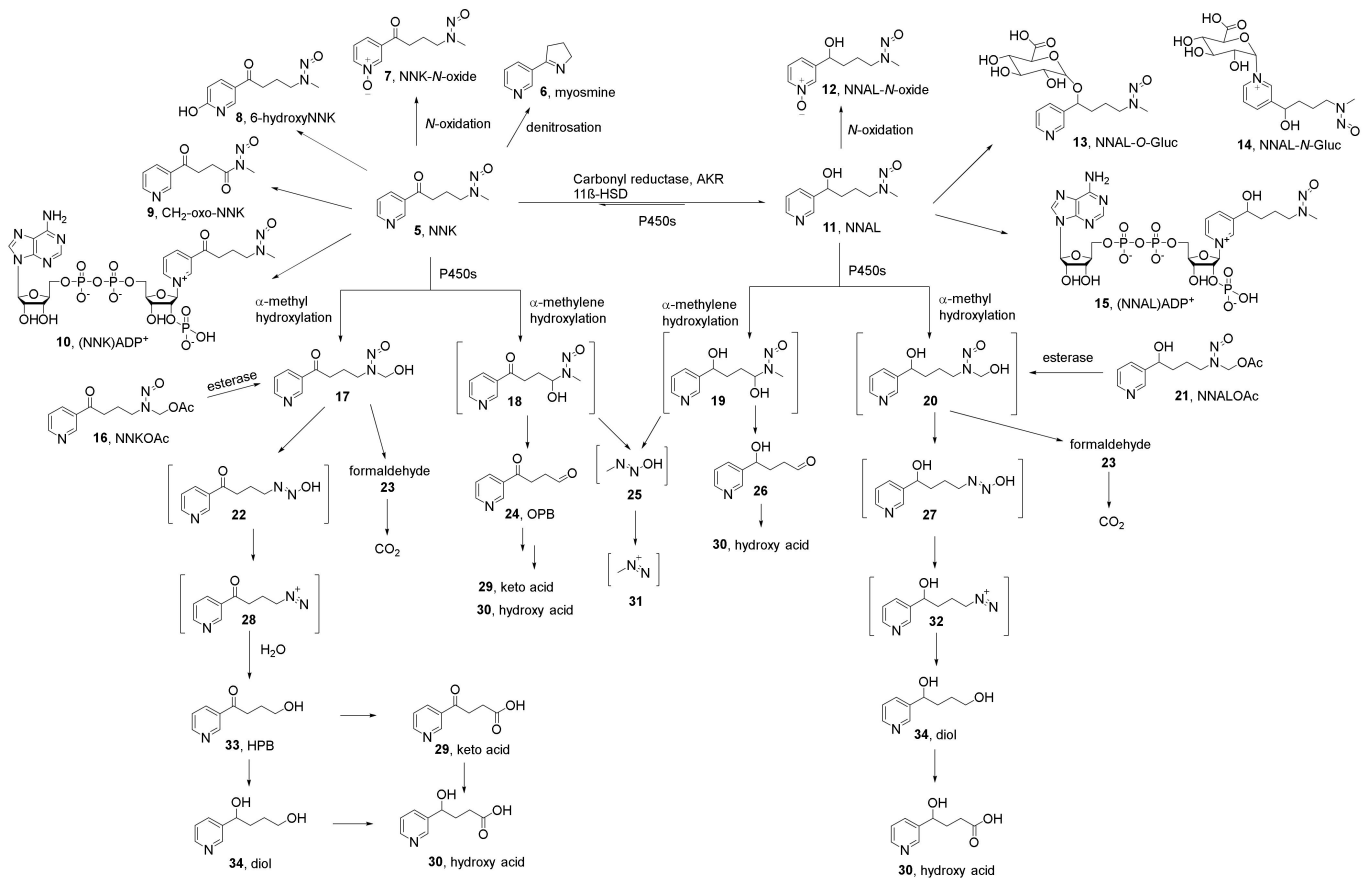
Mechanisms of NNK and NNAL metabolism.

Laboratory animal studies clearly demonstrate that NNK is a strong carcinogen, with a preference for inducing tumors of the lung [20]. NNK causes lung tumors in mice, rats and Syrian golden hamsters regardless of the administration pathway, including i.p. injection, s.c. injection, oral dosing in drinking water and oral swabbing [6,21]. NNK also induces tumors in the nasal cavity, liver and pancreas of rats [6]. Rat pancreatic tumors resulted from the metastasis of lung cancer induced by NNK and its metabolite 4-(methylnitrosamino)-1-(3-pyridyl)-1-butanol **11** (NNAL, Scheme 2) [22]. Pancreatic tumorigenesis in NNK-treated rats was enhanced and accelerated by dietary fat [23]. To evaluate the contribution of NNAL enantiomers to the carcinogenicity of NNK, a 90-week rat study with NNK, (*S*)- or (*R*)-NNAL was conducted. The three carcinogens were administered in the drinking water at concentrations of 5 ppm. Racemic NNAL was also tested at 10 ppm in the same study. The target organs were the lung and the pancreas. NNK, (*S*)-NNAL and (*R*)-NNAL induced lung tumors in nearly all the rats tested. NNK was significantly more carcinogenic than both the NNAL enantiomers (*p* < 0.001) based on the severity of the lung lesions. The difference between (*S*)-NNAL and (*R*)-NNAL was not statistically significant [24].

### 3.2. Metabolism

#### 3.2.1. Metabolic Activation and Detoxification Pathways

NNK is extensively bioactivated through multiple metabolic pathways (Scheme 2). The major metabolic activation pathways are α-hydroxylation (α-methyl and α-methylene) and carbonyl reduction, followed by α-hydroxylation, while *N*-oxidation and glucuronidation are considered as detoxification pathways [6].

The carbonyl reduction of NNK to NNAL is catalyzed by several enzymes, including carbonyl reductases, aldo-keto reductases (AKRs) and 11β-hydroxysteroid dehydrogenase type 1 (11β-HSD1) [25]. This conversion occurs predominately over the other metabolic pathways in nearly all studies in mice, rats, hamsters, patas monkeys and humans [26,27]. In rat pulmonary cells in vitro, carbonyl reduction predominated in all the tested cell types [28]. In A/J mouse lung explants, ~60% of NNK was converted to NNAL after 24 h incubation [29]. In explanted human tissues, NNAL constituted over 90% of the amount of radioactivity from NNK [30]. However, ~10–20% of NNAL is oxidized back to NNK by P450s in A/J mice and rats [31,32,33]. The administration pathways affect the metabolic conversion rates of NNK to NNAL. The conversion appeared to be more efficient via nose-only inhalation compared to i.p. injection and oral gavage [34]. Comparative carcinogenicity studies in A/J mice indicated that NNAL is a strong carcinogen comparable to or slightly weaker than NNK [29,35,36]; similar results were obtained in the carcinogenicity study in rats noted above [24]. Qualitatively and quantitatively similar results were also observed in the levels of DNA and hemoglobin adduct formation in rats treated with NNK and NNAL [31].

The primary bioactivation mechanism of NNK and NNAL is thought to be αhydroxylation. Both NNK and NNAL can be oxidized at the carbons adjacent to the nitroso group, catalyzed primarily by P450 2A13, which is principally expressed in the human lung and nasal cavity [35]. For NNK α-methyl hydroxylation, the intermediate α-hydroxymethylNNK **17** (Scheme 2) is formed and can be detected in a stable form as its *O*-glucuronide [36]. It generates two intermediates—formaldehyde (**23**) and the unique diazohydroxide **22**. Formaldehyde can be further converted to formic acid and, ultimately, carbon dioxide; diazohydroxide **22** can form the highly reactive species pyridyloxobutyl (POB) diazonium ion **28**, which pyridyloxobutylates DNA. For NNK α-methylene hydroxylation, two intermediates, 4-oxo-4-(pyridin-3-yl)butanal **24** (OPB) and methane diazohydroxide **25**, are formed. The aldehyde intermediate **24** can be further converted to keto acid **29**, which is reduced to hydroxy acid **30**. The methane diazohydroxide intermediate **25** spontaneously loses one molecule of H_2_O and generates the alkylating species methyldiazonium ion **31**, which attacks DNA and forms methyl DNA adducts. Similarly, for the α-methyl hydroxylation of NNAL, formaldehyde and the unique pyridylhydroxybutyl (PHB) diazonium ion **32** are formed, with the latter forming PHB DNA adducts that are specific to NNK/NNAL exposure. The α-methylene hydroxylation of NNAL generates the same methyldiazonium ion **31** as is formed from NNK. The other metabolite, 4-hydroxy-4-(pyridine-3-yl)butanal (**26**), can be further metabolized to hydroxy acid **30**. Clearly, α-hydroxylation is the major metabolic pathway to bioactivate NNK/NNAL to exert their carcinogenicity. The highly DNA-reactive electrophiles, including POB diazonium ion **28**, PHB diazonium ion **32**, methyldiazonium ion **31** and, probably, the aldehydes, such as formaldehyde **23** and OPB **24**, are considered responsible for the mutagenicity and carcinogenicity of NNK [6,37].

The *N*-oxidation of the pyridine ring of NNK/NNAL also occurs in vivo. The enzyme responsible for catalyzing the formation of pyridyl-*N*-oxides **7** and **12** is not fully characterized. P450 2B1 is the principal responsible enzyme in rat livers but not the lungs; P450 3A4 is suggested to be associated with the formation of the *N*-oxides of NNK and NNAL in the human liver [6]. NNK-*N*-oxide is significantly less tumorigenic than NNK and NNAL in mice, and is thus considered a detoxification metabolite [29]. The levels of NNK-*N*-oxide and NNAL-*N*-oxide have been quantified in human urine samples. While NNK-*N*-oxide was not detected in smokers’ urine, NNAL-*N*-oxide occurred at 0.53 ± 0.36 and 0.41 ± 0.35 pmol/mg creatinine in the urine of smokers and smokeless tobacco users, respectively. This indicates that the pyridine-*N*-oxidation metabolites of NNK/NNAL only represent a relatively minor detoxification pathway of NNK/NNAL metabolism in humans [38].

The glucuronidation of NNAL represents one of the most important detoxification pathways of NNK/NNAL metabolism. In 1993, we first reported the detection of NNAL glucuronides in the urine of smokers [39]. These metabolites were later widely detected in human samples, such as urine, blood, saliva and amniotic fluid [40,41,42,43,44,45,46,47]. Two glucuronides of NNAL have been identified: 4-(methylnitrosamino)-1-(3-pyridyl)-1-(*O*-β-D-glucopyranuronosyl)butane **13** (NNAL-*O*-Gluc) and 4-(methylnitrosamino)-1-(3-pyridyl-*N*-β-D-glycopyranuronosyl)-1-butanolonium inner salt **14** (NNAL-*N*-Gluc). NNAL-*N*-Gluc comprised 50% of the total NNAL-glucuronides in the urine of smokers and 24% in the urine of snuff-dippers [48]. The average level of total NNAL (free NNAL plus NNAL glucuronides) in the urine of 2641 smokers was 1.65 ± 2.13 pmol/mL, of which NNAL-*O*-Gluc and NNAL-*N*-Gluc accounted for 48 ± 15% and 22 ± 14% of the total NNAL, respectively [49].

NNK and NNAL are also biotransformed through some minor pathways. Hydroxylation occurring on the pyridine ring of NNK leads to the formation of 4-(methylnitrosamino)-1-(3-(6-hydroxypyridyl))-1-butanone **8** (6-hydroxyNNK), which is a minor metabolite representing approximately 1% of the urinary metabolites in NNK-treated rats and mice [50]. Nicotinamide adenosine dinucleotide phosphate (NADP) analogs of NNK (**10**, (NNK)ADP^+^) and NNAL (**15**, (NNAL)ADP^+^) were observed in the incubation mixtures with rat liver and pancreatic microsomes. They were suggested to be products of the transglycosylation reactions catalyzed by microsomal NAD^+^ glycohydrolases [51]. The denitrosation of NNK, a putative detoxification pathway, was observed with the release of nitrite in rat liver microsomal incubations [52]. The decomposition products of NNK denitrosation were proposed to be OPB **24** and myosmine **6** by analogy to the α-radical denitrosation mechanism of NDMA [53]. A new P450-mediated oxidation metabolite, 4-(methylnitrosamino)-1-(3-pyridyl)-1,4-butanedione (**9**, CH_2_-oxo-NNK), was detected in vitro with low conversion rates and catalyzed by P450 2A13 [54]. It can methylate DNA in vitro. No formation of 4-(nitrosoformamido)-1-(3-pyridyl)-1-butanone (CH_3_-oxo-NNK) was observed in the same incubation mixture, possibly due to its relatively short half-life (6.7 min) compared to that of the CH_2_-oxo-NNK (35.5 min) [54].

The end metabolites of NNK are primarily keto acid **29** and hydroxy acid **30**. In F344 rats, over 99% of gavaged NNK was metabolized in 48 h. The major ultimate metabolite was keto acid (38%), followed by hydroxy acid (14%), NNAL (10%) and NNK-*N*-oxide (3%) [55]. In male hamsters given s.c. injections of NNK, the major end metabolites in 48-h urine samples were hydroxy acid (16.8%), keto acid (8.4%), NNAL (6.9%). No NNK-*N*-oxide was detected [56]. In 20 smokers who smoked cigarettes containing [pyridine-*d*_4_]NNK for one week, ~86% of NNK was metabolically activated, with [pyridine-*d*_4_]keto acid and [pyridine-*d*_4_]hydroxy acid as the two principal end metabolites in the urine. The [pyridine-*d*_4_]keto acid contributed an average of 28.2% to the sum of these two metabolites [57]. However, in a second study, [pyridine-*d*_4_]hydroxy acid was found at an average level of 130 (range: 25–390) fmol/mL, lower than in the first study, in the urine of 87 smokers who smoked cigarettes spiked with [pyridine-*d*_4_]NNK for 1 week [58]. The high reduction rate of keto acid to hydroxy acid in humans (85%) was in sharp contrast to that in rats (~1%) [59,60].

#### 3.2.2. Stereochemistry of NNAL and Its Glucuronides

**Scheme 3:**
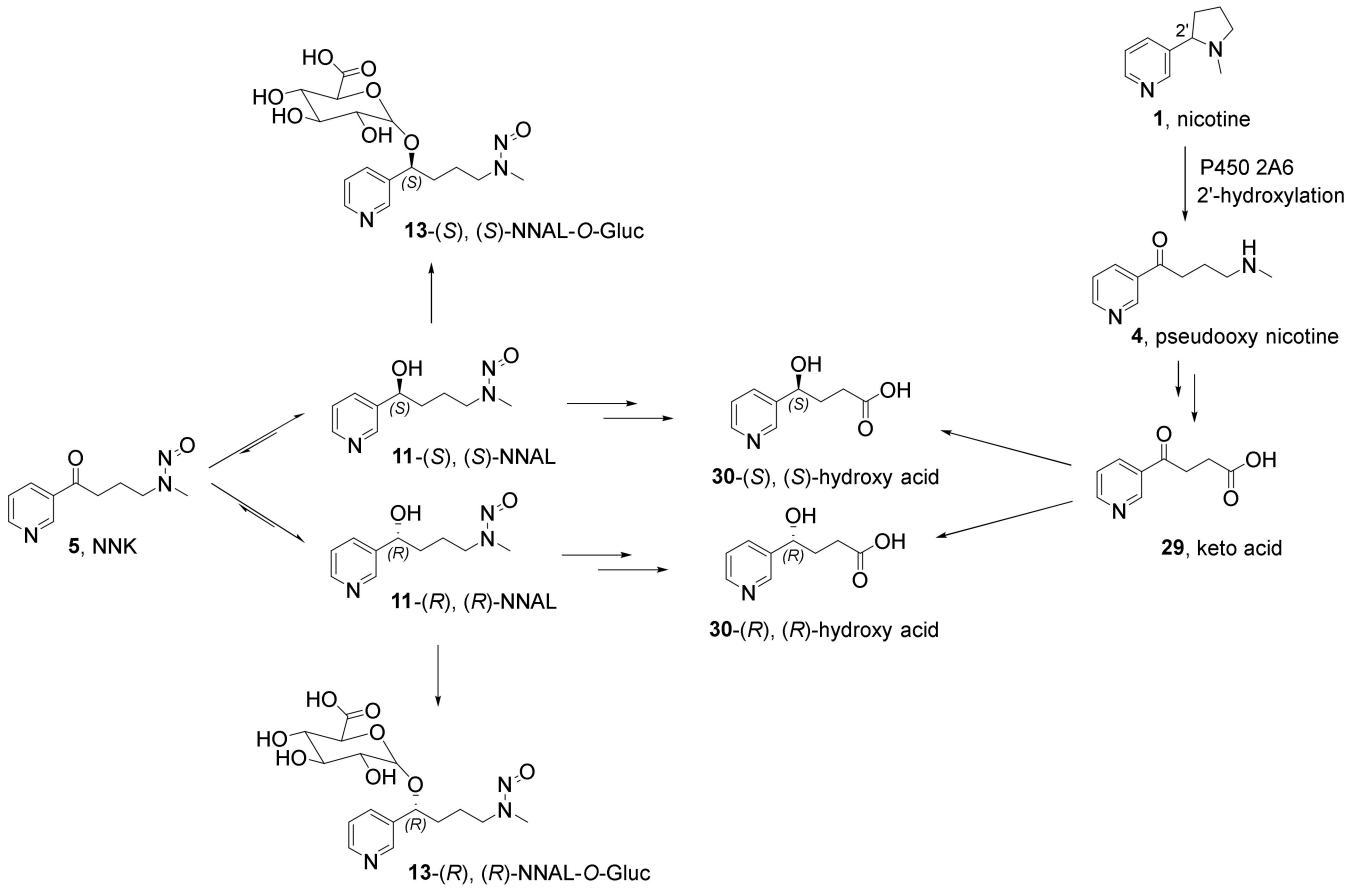
Stereochemistry of the formation of NNAL and its glucuronides.

Due to the chiral δ-carbon (carbinol carbon), there are two NNAL enantiomers—(*S*)-NNAL (**11**-(*S*)) and (*R*)-NNAL (**11**-(*R*)) (Scheme 3). The stereochemical aspects of NNAL formation by NNK metabolism have been investigated. In rodent tissues, including microsomes and cytosol from male F344 rat livers and lungs and female A/J mouse livers and lungs, (*S*)-NNAL predominated at 90–98% of NNAL formed by NNK. In human tissues, including liver microsomes, liver cytosol and red blood cells, (*S*)-NNAL predominated at 64, 90 and >95% of NNAL, respectively [61]. The preferential formation of (*S*)-NNAL over its (*R*)-counterpart is largely due to the high enantioselectivity of NNK reductases [25]. The aldo-keto reductases (AKRs) and carbonyl reductase purified from human liver cytosol produced >90% (*S*)-NNAL, in contrast to the formation of 35% (*R*)-NNAL by 11β-hydroxysteroid dehydrogenase type 1 (11β-HSD1) purified from human liver microsomes. Interestingly, human lung and placenta microsomes catalyzed NNK to NNAL with high selectivity to form (*R*)-NNAL, with a production of 89% and 87% of total NNAL, respectively [25]. The metabolism of (*S*)- and (*R*)-NNAL appears not to be enantioselective in human liver microsomes, even though some selectivity was observed in rat lung microsomes, where (*S*)-NNAL was preferentially oxidized to its metabolites. Both NNAL enantiomers were metabolized at similar rates by all the oxidative pathways in human liver microsomes [61].

The enantiomeric distribution of NNAL is intriguing. (*R*)-NNAL predominated in the bile or urine of rats treated with either NNK or NNAL. However, (*S*)-NNAL was significantly retained in the lungs of rats, comprising an (*S*)-/(*R*)-ratio of >20 after 24 h administration of racemic NNAL [62]. (*S*)-NNAL was also retained in humans, suggesting the existence of an unknown receptor for this enantiomer, possibly in the lungs [63]. (*S*)-NNAL accounted for 54% of NNAL enantiomers in the urine of human smokers (note that the original assignment of the stereochemistry of the two NNAL enantiomers was reversed. The corrected information was published in a 2000 corrigendum) [64,65].

The diastereomeric distribution of the NNAL glucuronide NNAL-*O*-Gluc has also been investigated. After the chiral derivatization with Mosher’s reagent, stereochemical configurations of the two NNAL-*O*-Gluc diastereomers were established [65,66]. (*S*)-NNAL-*O*-Gluc predominated in rat urine, while (*R*)-NNAL-*O*-Gluc was the major diastereomer in the bile of rats treated with either NNK or NNAL [62,67]. In the urine of patas monkeys and humans, (*S*)-NNAL-*O*-Gluc was the predominant diastereomer [26,66,67,68]. For example, it represented 68% of urinary NNAL-*O*-Gluc in cigarette smokers [64,65].

The stereochemical studies of NNK and NNAL metabolism may provide some explanations for the different carcinogenicities of NNAL enantiomers. In a study in A/J mice, (*S*)-NNAL showed equal lung tumorigenicity (25.6 ± 7.5 tumors/mouse) to NNK (25.3 ± 9.8 tumors/mouse), and was significantly more tumorigenic than racemic NNAL (12.1 ± 5.6 tumors/mouse) or (*R*)-NNAL (8.2 ± 3.3 tumors/mouse) [32]. The underlying mechanism was considered to be the preferential pulmonary metabolic activation of (*S*)-NNAL and its less effective glucuronidation than that of (*R*)-NNAL. However, an interspecies difference was observed in our chronic drinking water study with F344 rats. In this study, (*S*)-NNAL was equally potent to (*R*)-NNAL in causing lung and pancreatic tumors [24].

#### 3.2.3. Stereochemistry of Hydroxy Acid Formation

Similar to NNAL, hydroxy acid is comprised of two enantiomers due to the chiral δ-carbon (Scheme 3). In F344 rat urine, NNK metabolism produces mainly (*R*)-hydroxy acid (**30**-(*R*)), whereas nicotine metabolism generates the predominant (*S*)-hydroxy acid (**30**-(*S*)) [60,65]. This led to the hypothesis that the ratio of (*R*)-/(*S*)-hydroxy acid could be a biomarker specific to NNK metabolism. In F344 rats, hydroxy acid accounted for 12% of the NNK dose, in sharp contrast to the 0.1% of the nicotine dose [60]. This difference was considered sufficiently great to distinguish the origin of (*R*)-hydroxy acid from NNK metabolism versus from a minor 2′-hydroxylation metabolic pathway of nicotine metabolism via the intermediate **4** [69,70]. High stereoselectivity in the formation of hydroxy acid has been suggested due to the highly enantioselective reduction process catalyzed by carbonyl reductase [68].

However, the hypothesis based on the use of the ratio of (*R*)-/(*S*)-hydroxy acid as a urinary biomarker does not hold true in humans. The conversion of nicotine to hydroxy acid is a substantial metabolic pathway in humans, accounting for ~5–6% of the estimated nicotine dose [59,71]. Even though the stereochemistry of urinary hydroxy acid is essentially the same—97% of (*S*)-hydroxy acid in nicotine-treated rats and 98.7% of (*S*)-hydroxy acid in human nicotine-patch users [59,65]—the substantially high conversion rate of nicotine to hydroxy acid still makes it impossible to distinguish the origin of (*R*)-hydroxy acid in human urine due to the overwhelming abundance of nicotine (16.2–26.3 mg/g) in cigarette tobacco [72,73].

### 3.3. DNA Adducts Formed by NNK Metabolism

**Scheme 4:**
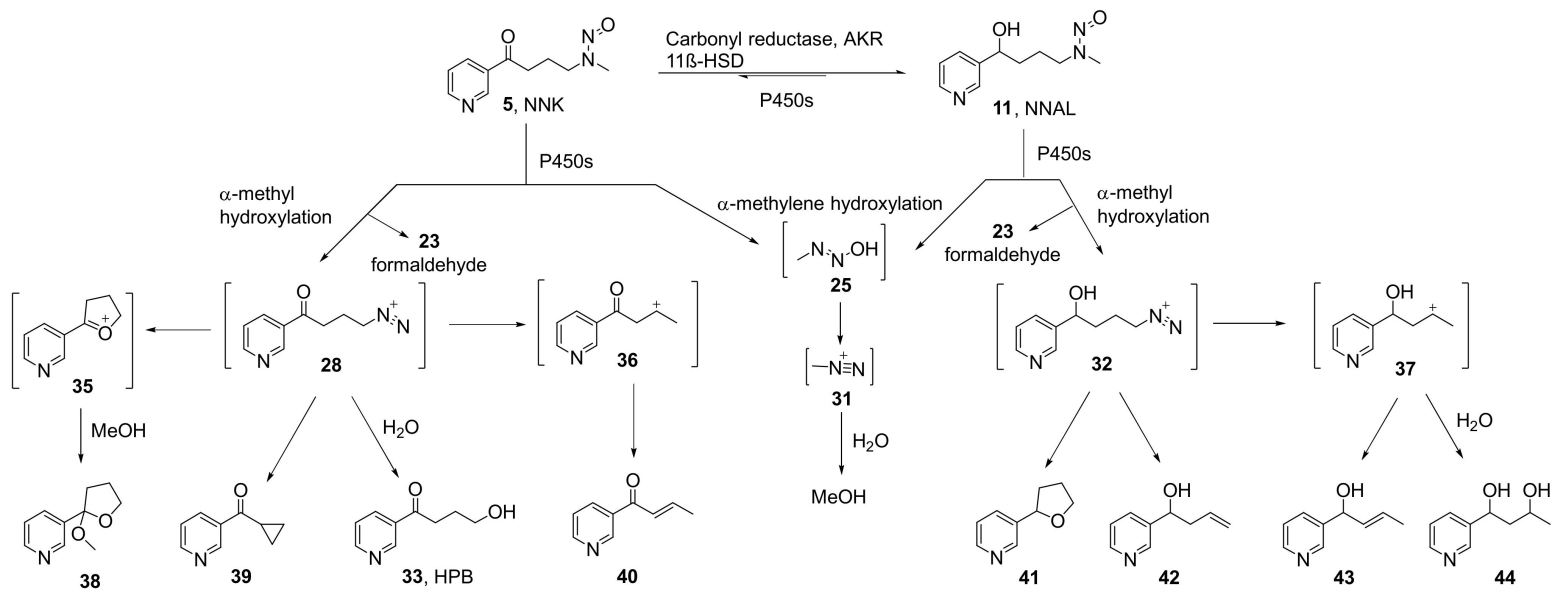
DNA-reactive intermediates formed by NNK and NNAL metabolism.

As shown in Scheme 4, NNK, via α-methyl hydroxylation, generates the POB diazonium ion **28**, which is highly reactive and pyridyloxobutylates DNA. The presence of compounds **33** and **39** as solvolysis products provides solid evidence for its formation [74]. The POB diazonium ion also rearranges and forms two secondary products—cyclic oxonium ion **35** and β-carbocation **36**—as demonstrated by the solvolysis product **38** and the dehydrogenation product **40**, respectively [74]. After the enzymatic reduction of NNK, the major metabolite NNAL generates the PHB diazonium ion **32** in a similar manner, via the α-methyl hydroxylation pathway. This results in the products **41** and **42**, which provide the evidence for its formation. The PHB diazonium ion attacks DNA and forms PHB DNA adducts. It also rearranges to the corresponding β-carbocation **37**, which has been suggested by the formation of the internal olefin **43** and the 1,3-diol **44** [74]. Formaldehyde **23** is also formed as a byproduct from the α-methyl hydroxylation of NNK and NNAL. Through the α-methylene hydroxylation pathway, both NNK and NNAL generate the methyldiazonium ion **31**.

The major DNA adducts formed by NNK metabolism can be generally classified as POB DNA adducts, PHB DNA adducts and methyl DNA adducts. Each class comprises DNA base adducts and phosphate adducts. The quantitation of several DNA adducts has shown their persistence and accumulation in the target tissues of laboratory animals, suggesting their potential as metabolic activation biomarkers of chronic NNK exposure in human tobacco users.

#### 3.3.1. POB DNA Adducts

(1) HPB-releasing DNA Adducts

**Figure 1:**
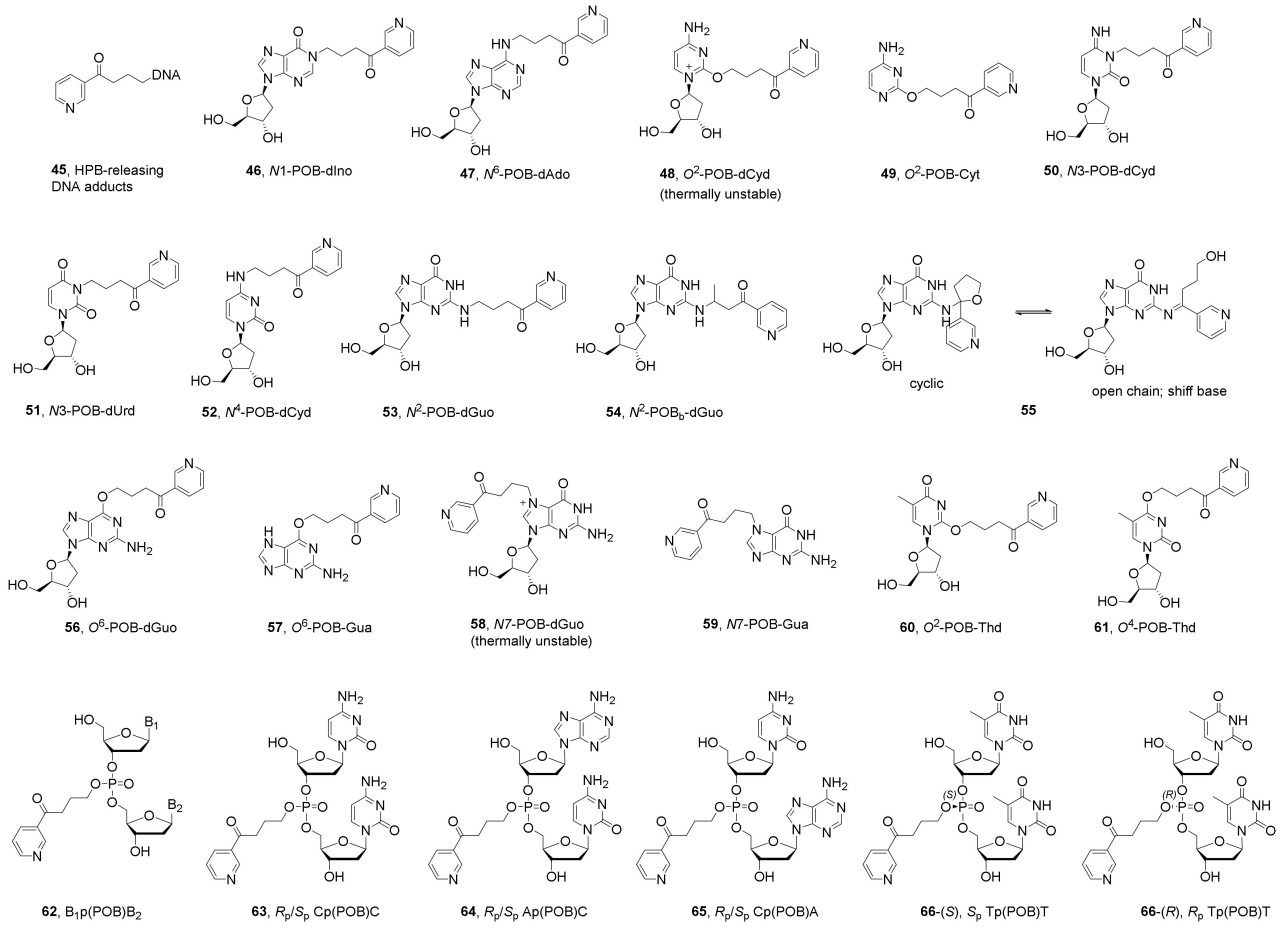
Structures of POB DNA base and phosphate adducts.

The POB DNA adducts formed by [^3^H]NNK in the liver and lung DNA of F344 rats were first demonstrated by the release of [^3^H]4-hydroxy-1-(3-pyridyl)-1-butanone ([^3^H]HPB) upon the acid hydrolysis or neutral thermal hydrolysis of the DNA [75]. [^3^H]HPB accounted for over 50% of the radioactivity in hepatic DNA [75]. The decomposition of the HPB-releasing DNA adducts **45** (Figure 1) in vitro and in vivo was multiphasic, with detectable levels up to 4 weeks post-s.c.-injection in F344 rats [76]. In male F344 rats, after 4 days of consecutive i.p. injections with NNK (15–5000 μg/kg/day), 18–3400 and 58–2180 fmol HPB/mg DNA were released from rat liver and lung DNA, respectively (Table 1) [77]. HPB-releasing adducts were also quantified in the lung DNA of rats chronically treated with NNK or each of the NNAL enantiomers for up to 70 weeks [24]. The levels of HPB-releasing adducts peaked at 9 ± 3 pmol/mg DNA at weeks 10 and 30 in the NNK treatment group and decreased slightly, to 5 ± 2 pmol/mg DNA, at week 70. In the (*S*)-NNAL-treated rats, the levels of HPB-releasing adducts in the lung DNA peaked at 11 ± 3 pmol/mg DNA at week 50 and decreased to 5 ± 1 pmol/mg DNA at week 70. The total level of HPB-releasing adducts in the lung DNA of rats in the (*R*)-NNAL treatment group remained low throughout the 70-week study course, ranging from 1 to 2 pmol/mg DNA [24].

Even though HPB can be formed by the reaction of myosmine **6** (Scheme 2), a minor tobacco alkaloid and dietary component, with NaNO_2_ under acidic conditions in vitro, HPB-releasing DNA adducts were not detected in rats treated with a combination of myosmine plus NaNO_2_ [78].

**Table 1:** Levels of HPB-releasing adducts quantified in laboratory animal and human tissues.

Animal Data							
Animal Species	Administration Pathway	Exposure Amount		Exposure Duration	Target Tissue DNA	HPB-Releasing Adducts ^a^	Ref.
Female A/J mice	Single i.p. injection	10 μmol/mouse [5-^3^H]NNK		12–144 h	Lung	8.4 pmol/μmol Gua @ 24 h time point	[79]
Male F344 rats	Once daily i.p. injection	15–5000 μg/kg/day of [5-^3^H]NNK or [C^3^H_3_]NNK		4 days	Liver	18–3400 fmol/mg DNA	[77]
					Lung	58–2180 fmol/mg DNA	
	Single s.c. injection	2 μmol/rat [5-^3^H]NNK		24 h	Liver	0.67 ± 0.1 pmol/mg DNA	[75]
					Lung	Present	
	Single s.c. injection	0.81 mg/kg of [5-^3^H]NNK		24 h	Liver	2.1 ± 0.1 pmol/μmol Gua	[76]
					Lung	1.6 pmol/μmol Gua	
	In the drinking water	5 ppm NNK		10 weeks	Lung	9 ± 3 (pmol/mg DNA)	[24]
				30 weeks		9 ± 3	
				50 weeks		6 ± 1	
				70 weeks		5 ± 2	
		5 ppm (*S*)-NNAL		10 weeks	Lung	5 ± 3 (pmol/mg DNA)	
				30 weeks		10 ± 5	
				50 weeks		11 ± 3	
				70 weeks		5 ± 1	
		5 ppm (*R*)-NNAL		10 weeks	Lung	1 ± 0 (pmol/mg DNA)	
				30 weeks		1 ± 1	
				50 weeks		2 ± 1	
				70 weeks		1 ± 1	
Female A/J mice	Single i.p. injection	10 μmol/mouse [5-^3^H]NNN		24 h	Liver	22.6 (or 25.1) pmol/μmol Gua	[80]
		11 μmol/mouse [5-^3^H]NNN			Lung	5.6 pmol/μmol Gua	[79]
Male F344 rats	Single s.c. injection	0.35 μmol/rat [5-^3^H]NNN		24 h	Liver	0.08 ± 0.1 pmol/mg DNA	[75]
	Once daily i.p. injection	0.407 μmol/rat [5-^3^H]NNN		3 days	Liver	0.54 pmol/mg DNA	[81]
					Lung	0.50	
					Nasal mucosa	0.02	
					Esophagus	<0.005	
					Kidney	<0.005	
**Human Data**							
**Sample source**	**Smoking status**	**Subject number**	**Tissues**	**HPB-releasing adducts**	**Note**		**Ref.**
Autopsy samples	Smokers verified by blood nicotine and cotinine, and medical record if necessary	9	Tracheobronchus	16 ± 18 fmol/mg DNA			[80]
			Peripheral lung tissues	11 ± 16			
	Nonsmokers	8	Tracheobronchus	0.9 ± 1.7	Only 1 subject had significant HPB levels.		
			Peripheral lung tissues	0.9 ± 2.3			
TobPRAC Biorepository	Smokers (>10 cigs/day for at least 1 year) verified by urinary NNN and NNAL.	30	Mouthwash oral cells	12.0 ± 35.1 pmol/mg DNA			[82]
			Buccal cells	45 ± 57			
	Nonsmokers	15	Mouthwash oral cells	0.23 ± 0.43	Only 3 subjects had significant HPB levels.		
Patients with HNSCC	Smokers (determined by lifetime tobacco use questionnaire)	30	Buccal cells	8.19 ± 17.8 (median 1.51) pmol/mg DNA			[83]
Patients without HNSCC		35		4.53 ± 14.36 (median 0.23)			
Patients with lung cancers	Self-reported smokers	21	Peripheral lung tissues	404 ± 258 fmol/mg DNA			[84]
	Self-reported nonsmokers	11		59 ± 56			
Tumor-free sudden death victims	Smokers (>15 ng cotinine/mL blood or >100 ng cotinine/mL urine)	32	Lung	92 ± 148 fmol/mg DNA	Primarily road traffic accidents, suicide and sudden cardiac arrest.		[85]
		29	Mucosa of esophagus	138 ± 208			
		12	Mucosa of cardia	93.6 ± 91.9			
	Nonsmokers verified by blood or urinary cotinine	56	Lung	61 ± 66			
		53	Mucosa of esophagus	131 ± 130			
		18	Mucosa of cardia	117 ± 110			
Patients with or without upper gastrointestinal disorders	Self-reported smokers	7	Mucosal biopsies of the lower esophagus	4.80 ± 3.57 pmol/mg DNA	One outlier of patient #55 with ulcerative gastritis was excluded due to its exceptionally high level of HPB-releasing adducts (36.98 pmol/mg DNA)		[86]
	Self-reported nonsmokers	7		2.86 ± 2.44			

^a^ 1 pmol/mg DNA = 1.51 pmol/μmol Gua = 0.33 adducts/10^6^ nucleotides. These conversion factors were calculated based on 1 mg DNA containing approximately 3 μmol nucleotides, whereas dGuo accounted for ~22% of total nucleotides [87].

(2) POB DNA Base Adducts

In an effort to characterize the chemical structures of HPB-releasing DNA adducts, Peterson et al. found that Gua adducts predominated in the reaction mixture of 4-((acetoxymethyl)nitrosamino)-1-(3-pyridyl)-1-butanone **16** (NNKOAc, Scheme 2) with different polymers and oligomers [88]. *O*^6^-(4-(3-Pyridyl)-4-oxobut-1-yl)-2′-deoxyguanosine **56** (*O*^6^-POB-dGuo, Figure 1) was first characterized in calf thymus DNA reacted with NNKOAc as the deglycosylated form, *O*^6^-POB-Gua **57** [89]. The major HPB-releasing DNA adducts identified in vitro are two thermally unstable POB DNA base adducts, *N*7-(4-(3-pyridyl)-4-oxobut-1-yl)-2′-deoxyguanosine **58** (*N*7-POB-dGuo) and *O*^2^-(4-(3-pyridyl)-4-oxobut-1-yl)-2′-deoxycytidine **48** (*O*^2^-POB-dCyd) [90,91]. The stable form, *N*7-POB-Gua **59**, accounted for 30–35% of the total HPB-releasing adducts in the DNA treated by NNKOAc [90]. Other adducts that are thermally stable but still release HPB after acid hydrolysis have also been identified in vitro. These include *N*1-(4-(3-pyridyl)-4-oxobut-1-yl)-2′-deoxyinosine **46** (*N*1-POB-dIno), *N*^6^-(4-(3-pyridyl)-4-oxobut-1-yl)-2′-deoxyadenosine **47** (*N*^6^-POB-dAdo), *N*3-(4-(3-pyridyl)-4-oxobut-1-yl)-2′-deoxycytidine **50** (*N*3-POB-dCyd), *N*^4^-(4-(3-pyridyl)-4-oxobut-1-yl)-2′-deoxycytidine **52** (*N*^4^-POB-dCyd), *N*^2^-(4-(3-pyridyl)-4-oxobut-1-yl)-2′-deoxyguanosine **53** (*N*^2^-POB-dGuo), *N*^2^-(2-(3-pyridyl)tetrahydrofuran-2-yl)-2′-deoxyguanosine, or its open chain tautomer (Schiff base) **55**, *O*^6^-POB-dGuo (**56**), *O*^2^-(4-(3-pyridyl)-4-oxobut-1-yl)thymidine **60** (*O*^2^-POB-Thd) and *O*^4^-(4-(3-pyridyl)-4-oxobut-1-yl)thymidine **61** (*O*^4^-POB-Thd) (Figure 1) [90,91,92,93,94]. A unique dGuo adduct formed by NNKOAc has been identified as *N*^2^-(4-(3-pyridyl)-4-oxobut-2-yl)-2′-deoxyguanosine **54** (*N*^2^-POB_b_-dGuo). This adduct was hypothesized to be formed by the rearranged carbocation **36**, or the corresponding α,β-unsaturated ketone **40** (Scheme 4) [95].

It is important to note that many adducts identified in vitro have not been detected in vivo. For example, *N*1-POB-dIno **46** was readily formed in NNKOAc-treated calf thymus DNA but was not detected in rat liver or lung DNA [93]. The Schiff base adduct **55** was only detected in relatively small amounts in the reaction of NNKOAc and dGuo in its reduced form [90]. *N*3-POB-dCyd **50** and *N*^4^-POB-dCyd **52** were formed as minor products in vitro. *N*3-POB-dCyd was found to easily undergo deamination and form *N*3-(4-(3-pyridyl)-4-oxobut-1-yl)-2′-deoxyuridine **51** (*N*3-POB-dUrd) under HPLC purification conditions [92]. The unique *N*^2^-POB_b_-dGuo adduct **54** was not detected in the DNA of rats treated with NNK or NNN [81].

The distribution patterns of POB DNA base adducts in vitro differ greatly from those in vivo. In NNKOAc-treated calf thymus DNA, the relative amounts of the POB DNA adducts were: *N*7-POB-Gua **59** > *O*^6^-POB-dGuo **56** > *O*^2^-POB-Thd **60** > *O*^2^-POB-Cyt **49** [96]. In the lung DNA of mice administered NNK by a single i.p. dose, *N*7-POB-Gua was the most abundant POB DNA base adduct, followed by *O*^2^-POB-Thd, at slightly lower concentrations. *O*^6^-POB-dGuo was substantially lower than *O*^2^-POB-Thd. The lowest adduct concentration was that of *O*^2^-POB-Cyt [97]. In the liver and lung DNA of rats administered 0.025 or 0.1 mmol/kg body weight of NNK by s.c. injection for 4 consecutive days, *N*7-POB-Gua and *O*^2^-POB-Thd predominated among the four quantified adducts (Table S1). The lowest DNA adduct concentration was that of *O*^6^-POB-dGuo [96]. In a 20-week NNK drinking-water study in rats, *O*^2^-POB-Thd predominated among the POB DNA adducts detected in rat livers, lungs, nasal respiratory mucosa, nasal olfactory mucosa, oral mucosa and pancreas at each time point. *O*^6^-POB-dGuo was only detected in the lung DNA at very low levels [98,99]. A similar trend was observed in our chronic carcinogenicity study of NNK in F344 rats. Throughout the study course of up to 70 weeks, *O*^2^-POB-Thd was the most abundant POB DNA base adduct at each time point in NNK-treated rat tissues. *O*^6^-POB-dGuo was marginally detectable at very low concentrations [24]. It is noteworthy that the persistence of the POB DNA base adducts varied. For example, the *O*^2^-POB-Thd peaked at week 30 with a maximum level of 4809 ± 193 fmol/mg rat lung DNA, while the *N*7-POB-Gua and the *O*^6^-POB-dGuo peaked at week 10, with maximum levels of 970 ± 148 and 34 ± 21 fmol/mg rat lung DNA, respectively [24].

In the same chronic rat study, we also investigated the carcinogenicity of (*S*)-NNAL and (*R*)-NNAL. The DNA adducts formed by these two NNAL enantiomers were quantified. The POB DNA base adducts were formed by both enantiomers. However, the concentrations of these POB DNA base adducts were strikingly different. (*S*)-NNAL produced significantly higher levels of *O*^2^-POB-Thd, *N*7-POB-Gua and *O*^6^-POB-dGuo than the (*R*)-NNAL in all the examined rat tissues at each time point (Table S1). The levels of these three POB DNA base adducts were comparable in the (*S*)-NNAL treatment group and the NNK treatment group [24]. These results were also consistent with the data from our 20-week rat study with (*S*)- or (*R*)-NNAL [98,99]. The difference in the formation of POB DNA base adducts by (*S*)-NNAL and (*R*)-NNAL has been proposed to result from (1) the preferential persistence of (*S*)-NNAL and (2) the higher re-oxidation rates of (*S*)-NNAL to NNK in the target tissues, such as those of the lungs [98]. Through re-oxidation, NNAL is converted back to NNK, which undergoes the α-methyl hydroxylation pathway, and forms the corresponding POB DNA base adducts.

(3) POB DNA Phosphate Adducts

The phosphate groups in the DNA backbone have free hydroxy groups that can be alkylated by NNK metabolites. Due to the chirality of the phosphorus atom, the POB DNA phosphate adduct **62** (3′ → 5′, Figure 1), if formed, will comprise two or four diastereomers, depending on whether the nucleobases connected to the phosphate group are the same or different. There are up to 10 combinations of nucleobases and, thus, up to 32 diastereomers of POB DNA phosphate adducts that can be formed by DNA phosphate pyridyloxobutylation [100].

In 2002, Tornqvist et al. reported the first evidence of DNA phosphate adduct formation by NNK. The total level of POB DNA phosphate adducts accounted for ~22% of the total pyridyl DNA adducts. However, no structural information was provided using their cob(I)alamin transformation method [101]. We later characterized the chemical structures of POB DNA phosphate adducts using high-resolution mass spectrometry. Up to 30 NNK-derived POB DNA phosphate adducts were characterized in the calf thymus DNA treated with NNKOAc. All the 10 combinations of POB DNA phosphate adducts were detected in the liver and lung DNA of rats treated with NNK [102]. The concentrations of POB DNA phosphate adducts were quantified using the same rat tissues from our chronic carcinogenicity study (Table S1) [24]. The total POB phosphate adducts accounted for 5–9% of the total POB DNA adducts, ranging 89–190 and 218–475 fmol/mg DNA in the rat livers and rat lungs, respectively, over the 70-week study course [103].

The POB DNA phosphate adducts were also formed by (*S*)-NNAL and (*R*)-NNAL in the tissues of the rats from the same chronic carcinogenicity study [104]. The (*S*)-NNAL caused significantly higher levels of POB DNA phosphate adducts than the (*R*)-NNAL in the rat lung DNA at each time point. The total POB DNA phosphate adducts in the (*S*)-NNAL treatment group were 1180–4650 fmol/mg lung DNA with the maximum level peaking at 10 weeks. In the (*R*)-NNAL treatment group, they occurred only at 46–175 fmol/mg lung DNA with no significant changes in the first 50 weeks, followed by decreasing levels at week 70 [104]. This was consistent with the POB DNA base adduct formation in both treatment groups. As discussed above, a high conversion rate of (*S*)-NNAL re-oxidation to NNK is considered to be responsible for this difference.

The distribution patterns of 10 combinations of POB DNA phosphate adducts were slightly different in the rat livers and lungs. However, adducts such as Cp(POB)C **63** (Figure 1), Ap(POB)C **64** or Cp(POB)A **65** and combinations with Thd appeared to be predominant [102]. Due to the chirality of the phosphorus atom, these adducts may comprise two or four diastereomers. The formation and persistence of the diastereomers were also different. For example, one diastereomer of Tp(POB)T (**66**-(*S*) or **66**-(*R*)) remained at low concentrations with a slowly decreasing trend throughout the 70-week study course, while the other diastereomer reached its maximum level at week 30 and remained at nearly the same level up to 70 weeks [102]. This is likely the balanced result of adduct formation and enzymatic repair in vivo. However, the stereochemical aspects of the repair mechanisms of POB DNA phosphate adducts are not well understood [105].

#### 3.3.2. PHB DNA Adducts

As illustrated by Scheme 4, NNK can be reduced to NNAL, which is metabolized to the PHB diazonium ion **32** and the rearranged carbocation **37**, which can react with DNA, forming the corresponding PHB DNA base and phosphate adducts.

(1) PHB DNA Base Adducts

**Figure 2:**
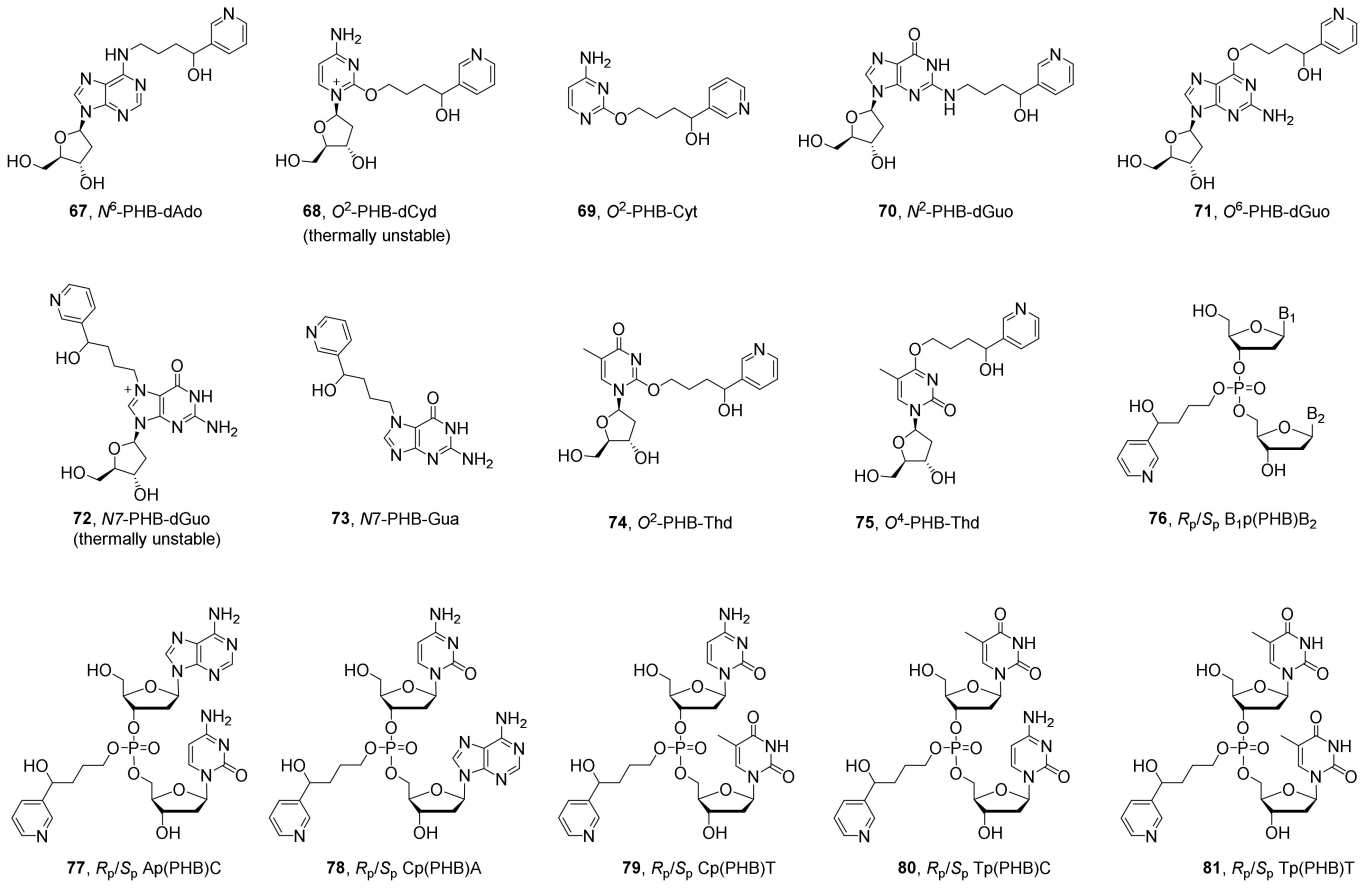
Structures of PHB DNA base and phosphate adducts.

To characterize the chemical structures of PHB DNA base adducts, a regiochemically activated form of NNAL—4-(acetoxymethylnitrosamino)-1-(3-pyridyl)-1-butanol **21** (NNALOAc, Scheme 2)—was reacted with dGuo and DNA in vitro. *N*7-(4-(3-Pyridyl)-4-hydroxybut-1-yl)-2′-deoxyguasosine **72** (*N*7-PHB-dGuo, Figure 2) and its stable product, *N*7-(4-(3-pyridyl)-4-hydroxybut-1-yl)guanine **73** (*N*7-PHB-Gua), together with *O*^6^-(4-(3-pyridyl)-4-hydroxybut-1-yl)-2′-deoxyguanosine **71** (*O*^6^-PHB-dGuo) and *N*^2^-(4-(3-pyridyl)-4-hydroxybut-1-yl)-2′-deoxyguanosine **70** (*N*^2^-PHB-dGuo), were unambiguously detected in these reactions [106]. When NNALOAc reacted with dCyd, Thd and DNA, the adducts to the pyrimidine rings were identified as *O*^2^-(4-(3-pyridyl)-4-hydroxybut-1-yl)cytidine **69** (*O*^2^-PHB-Cyt), the stable form of *O*^2^-PHB-dCyd **68** and *O*^2^-(4-(3-pyridyl)-4-hydroxybut-1-yl)thymidine **74** (*O*^2^-PHB-Thd) [91]. The dAdo-derived PHB adduct formed in vivo by NNK or NNAL was characterized as *N*^6^-(4-(3-pyridyl)-4-hydroxybut-1-yl)-2′-deoxyadenosine **67** (*N*^6^-PHB-dAdo) [93]. A minor adduct, *O*^4^-(4-(3-pyridyl)-4-hydroxybut-1-yl)thymidine **75** (*O*^4^-PHB-Thd), was also detected in the cellular genomic DNA exposed to NNALOAc in vitro [107].

In the target tissues of rats treated with 10 ppm NNK or each of the two NNAL enantiomers in the drinking water for 20 weeks, three major PHB DNA base adducts, *O*^2^-PHB-Thd **74**, *N*7-PHB-Gua **73** and *O*^6^-PHB-dGuo **71**, were quantified. As shown in Table S1, *O*^2^-PHB-Thd predominated among the three PHB DNA base adducts in all the rat tissues. The highest concentrations of *O*^2^-PHB-Thd were observed in the nasal respiratory mucosa of all three treatment groups. Similarly, *N*7-PHB-Gua and *O*^6^-PHB-dGuo were also found in their highest levels in the nasal respiratory mucosa in the NNK treatment group and the (*S*)-NNAL treatment group. However, the lung tissue contained nearly equal or even higher concentrations of the three PHB DNA base adducts compared with the nasal respiratory mucosa in the rats from the (*R*)-NNAL treatment group. More strikingly, the total PHB DNA base adducts formed in the (*R*)-NNAL treatment group exceeded, to a significantly greater extent, those from each of the other two groups [99,108]. This difference in PHB DNA base adduct distributions across the different treatment groups was consistent with that of the POB DNA base adducts formed by NNK or NNAL enantiomers. This was hypothesized to have been due to the different metabolic profile of (*R*)-NNAL, which has a slower conversion rate to NNK [108]. NNK- and (*S*)-NNAL-PHB DNA base adducts were also detected in the mitochondrial DNA in the lungs and livers of rats from the same study. The levels of total POB- and PHB-DNA base adducts in the lung mitochondrial DNA (mtDNA) were higher than those in the nuclear DNA (nDNA). No difference was observed in the liver DNA [109].

**Figure 3:**
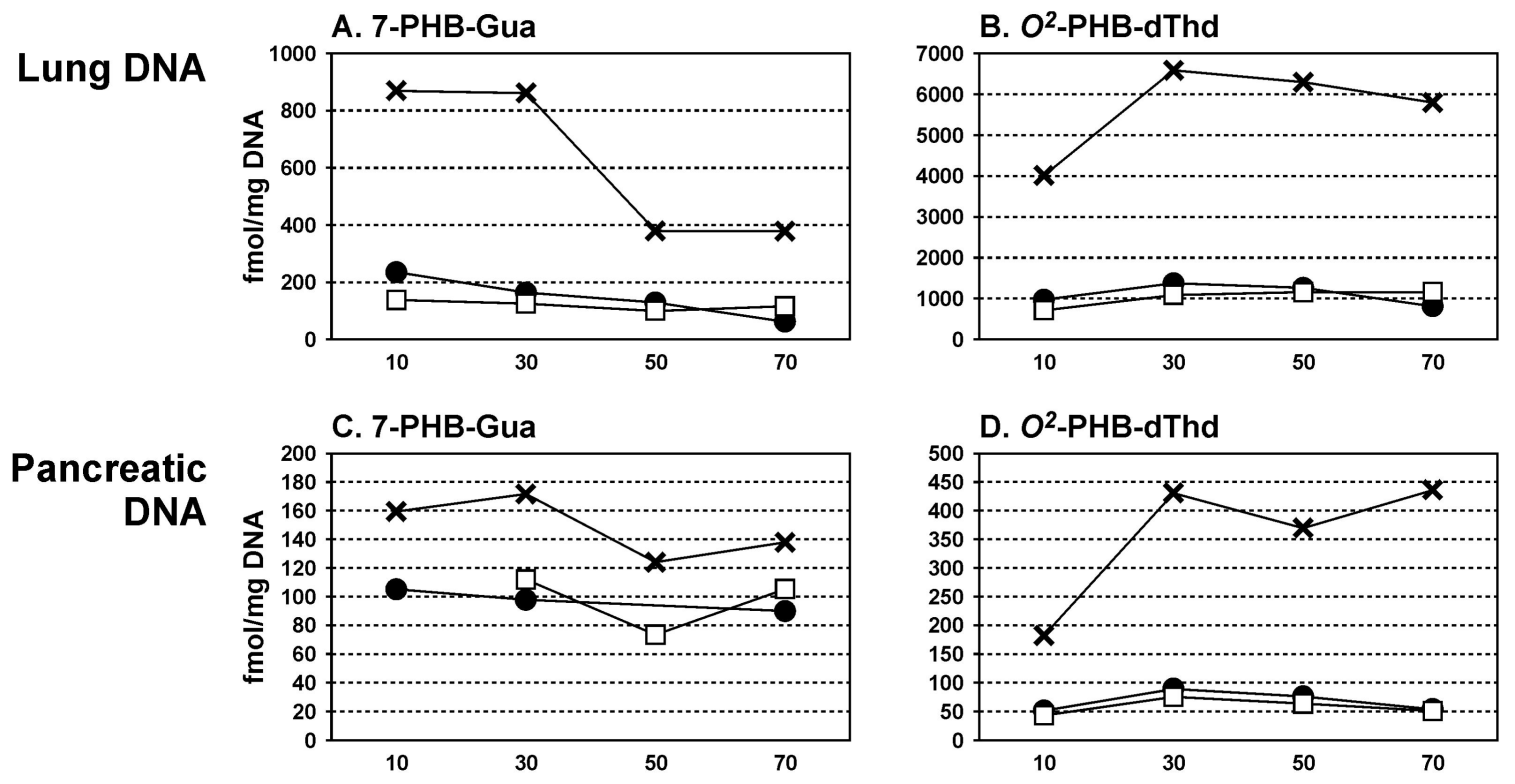
Levels of PHB DNA adducts in lung and pancreatic DNA of rats treated with 5 ppm NNK or NNAL enantiomers in the drinking water for 10, 30, 50, or 70 weeks. NNK: filled circle; (*S*)-NNAL: square; (*R*)-NNAL: cross. Adapted from ref. [24], with the permission of Oxford University Press.

In the lungs and pancreas (and liver, in some cases) of rats chronically administered NNK or (*S*)-NNAL or (*R*)-NNAL in drinking water for up to 70 weeks, the adduct-formation pattern was consistent with the 20-week rat study. As shown in Table S1, *O*^2^-PHB-Thd predominated among the three quantified adducts (*O*^2^-PHB-Thd, *O*^6^-PHB-dGuo and *N*7-PHB-Gua) in the rat lung DNA in all three treatment groups. It persisted at the highest concentrations of 4009–6581 fmol/mg DNA throughout the 70 weeks in the (*R*)-NNAL treatment group, while it still occurred at high levels of ~1000 fmol/mg DNA in the NNK or (*S*)-NNAL treatment group (Figure 3). A similar pattern was observed for *N*7-PHB-Gua, which persisted in significantly higher concentrations in the (*R*)-NNAL treatment group than in the other two. However, it did not persist throughout the 70-week study course, decreasing from ~860 to ~380 fmol/mg DNA at week 50. In the pancreatic DNA, both *O*^2^-PHB-Thd and *N*7-PHB-Gua persisted at significantly lower levels, but with a highly similar distribution pattern, as observed in the lung DNA [24]. The levels of *N*^6^-PHB-dAdo in the liver and lung DNA of rats treated by (*R*)-NNAL for 50 weeks (24 ± 2 and 125 ± 34 fmol/mg DNA, respectively) were also significantly higher than those in the same tissues of rats treated by NNK (6 ± 1 and 35 ± 5 fmol/mg DNA, respectively) or (*S*)-NNAL (5 ± 1 and 36 ± 8 fmol/mg DNA) [93].

(2) PHB DNA Phosphate Adducts

PHB DNA phosphate adducts were also quantified in the lung tissues of rats chronically treated with NNK, (*S*)-NNAL, or (*R*)-NNAL from the carcinogenicity study noted above (Table S1) [103,104]. A total of 107 structurally unique PHB DNA phosphate adducts **76** (Figure 2) were characterized in the lung DNA of rats treated with NNK [103]. In (*S*)- or (*R*)-NNAL-treated rats, the total PHB DNA phosphate adducts occurred at levels of 3480–4180 or 4530–6920 fmol/mg DNA throughout the 70-week study course. In the lungs of the NNK-treated rats, the total PHB DNA phosphate adducts occurred at levels of 3950–8160 fmol/mg DNA. In contrast to the occurrence of PHB DNA base adducts, the PHB DNA phosphate adducts did not display any significant difference across the three treatment groups. They accounted for 38–55, 30–36 and 45–51% of the total measured DNA adducts in the lungs of the rats in the treatment group of NNK, (*S*)-NNAL or (*R*)-NNAL, respectively. The diastereomeric differences were also similar to those observed for the POB DNA phosphate adducts. For example, some combinations, such as Tp(PHB)T **81**, Cp(PHB)T **79** or Tp(PHB)C **80**, Ap(PHB)C **77** or Cp(PHB)A **78** in the rat livers and Cp(PHB)T or Tp(PHB)C in the rat lungs, appeared to be the predominant adducts among all the PHB DNA phosphate adducts in the NNK-treatment group.

#### 3.3.3. Methyl DNA Adducts

(1) Methyl DNA Base Adducts

**Figure 4:**
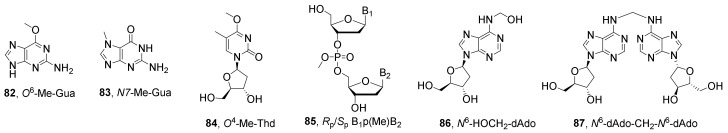
Structures of methyl DNA adducts and DNA adducts derived from formaldehyde.

*O*^6^-Methylguanine **82** (*O*^6^-Me-Gua, Figure 4) has been detected in target tissues—the nasal mucosa, lungs and livers—of F344 rats treated with NNK both acutely (4 h after a single i.v. injection of 87 mg/kg NNK) and chronically (daily i.p. injection of 40 mg/kg NNK for 14 days) [110]. Other methyl adducts, such as *N*7-methylguanine **83** (*N*7-Me-Gua) and *O*^4^-methylthymidine **84** (*O*^4^-Me-Thd), were also detected, with accumulation and persistence, in the respiratory tissues of rats repeatedly exposed to NNK via daily i.p. injections of 100 mg/kg for up to 12 days [111].

**Figure 5:**
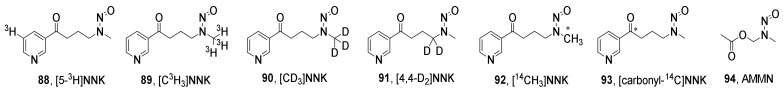
Structures of isotope-labeled NNKs and AMMN.

The levels of methyl DNA base adducts were greater than those of POB DNA base adducts in the livers and lungs of F344 rats administered [5-^3^H]NNK **88** (Figure 5) or [C^3^H_3_]NNK **89** by i.p. injection at 3–5000 μg/kg/day for 4 days. The levels of *N*7-Me-Gua were 0.22–246 pmol/μmol Gua in the rat liver DNA and 0.23–78.4 pmol/μmol Gua in the rat lung DNA. For comparison, the levels of released HPB from the same rat liver and lung DNA were 0–5.0 and 0–3.2 pmol/μmol Gua, respectively [77]. *O*^6^-Me-Gua was detected at its maximal level of 2550 ± 263 fmol/mg DNA at week 5 in the lung DNA of rats treated with 10 ppm NNK in drinking water for 20 weeks. Its levels were substantially higher than those of most of the POB- and PHB-DNA adducts, except for *O*^2^-POB-Thd and *N*7-POB-Gua [112]. We quantified the concentrations of *O*^6^-Me-Gua in the lung DNA of the rats from the chronic carcinogenicity study, which were administered 5 ppm NNK or NNAL enantiomers in their drinking water for up to 70 weeks (Table S1) [24]. In the lung DNA of the NNK- and (*S*)-NNAL-treated rats, *O*^6^-Me-Gua peaked at week 10 and decreased dramatically throughout the study course of 70 weeks, occurring at 213 ± 27 and 269 ± 178 fmol/mg DNA at weeks 10 and 34 and 49 ± 22 fmol/mg DNA at week 70. The levels of *O*^6^-Me-Gua in the lung DNA of rats from the (*R*)-NNAL treatment group were much lower, ranging from 17 to 4 fmol/mg DNA in the 70-week study course with no clear trend in changes. No such adduct was detected in the pancreas of rats from the same carcinogenicity study [24]. Considering the well-established miscoding properties of *O*^6^-Me-Gua, the discussion of its potential mutagenicity and carcinogenicity is presented in Section 3.5.

(2) Methyl DNA Phosphate Adducts

A methyl DNA phosphate adduct **85** (Figure 4) was also formed in the DNA of rats treated with NNK or each of the two NNAL enantiomers in our chronic carcinogenicity study [113]. It predominated in the lungs of the NNK-treated rats, occurring at 2290 ± 546 fmol/mg DNA at week 10 and increasing moderately to 4510 ± 129 fmol/mg DNA at week 70. The concentrations of the methyl DNA phosphate adducts were slightly lower in the rats treated with (*S*)-NNAL or (*R*)-NNAL. In the lungs of the (*S*)-NNAL-treated rats, the total methyl DNA phosphate adducts occurred at relatively stable levels of 872–1120 fmol/mg DNA across the 70-week study course. In the lungs of the (*R*)-NNAL-treated rats, these adducts occurred at 874 ± 146 fmol/mg DNA at week 10 and peaked at 1430 ± 140 fmol/mg DNA at week 30 and decreased to 763 ± 90 fmol/mg DNA at week 70 [113].

Among the 32 possible diastereomers of the methyl DNA phosphate adducts, a total of 23, 21 and 22 isomers in all 10 combinations were detected in the rat lung tissues. In the three treatment groups, the most abundant methyl phosphate adducts were Ap(Me)T or Tp(Me)A, Ap(Me)C or Cp(Me)A and Gp(Me)T or Tp(Me)G. Similar to the POB and PHB DNA phosphate adducts, diastereomeric differences in the formation and persistence of methyl DNA phosphate adducts were also clearly observed in the rat lung DNA at various time points [113].

#### 3.3.4. Formaldehyde-Derived DNA Adducts

Since formaldehyde is a metabolite formed by NNK and NNAL α-methyl hydroxylation, formaldehyde-derived DNA adducts were detected in vitro upon the hydrolysis of NNKOAc and NNALOAc [114]. These adducts were also detected in the DNA of rats treated with NNK. Substantial levels of *N*^6^-hydroxymethyl-2′-deoxyadenosine **86** (*N*^6^-HOCH_2_-dAdo, Figure 4) were detected in the liver and lung DNA, occurring at 3720 ± 2210 and 615 ± 504 fmol/mg DNA, respectively, when rats were dosed with 0.1 mmol/kg NNK by s.c. injection daily for 4 consecutive days. The levels of the cross-link adduct di-(*N*^6^-deoxyadenosyl)methane **87** (*N*^6^-dAdo-CH_2_-*N*^6^-dAdo) were significantly lower in the same samples, occurring at 303 ± 290 fmol/mg DNA in the rat livers and 18 fmol/mg DNA in the rat lungs [115].

#### 3.3.5. Summary of DNA Adducts Formed by NNK Metabolism

Three major types of DNA base adduct—POB DNA adducts, PHB DNA adducts and methyl DNA adducts—as well as the minor adducts derived from the NNK metabolite formaldehyde, have been well characterized. The formation and persistence of these DNA adducts in laboratory animals have been extensively studied (Table S1). In rats or mice treated acutely with NNK (from hours to a few days), *N*7-POB-Gua and *O*^6^-Me-dGuo were easily detected in relatively high abundance. However, their high concentrations did not persist for a long time, possibly due to spontaneous depurination (*N*7-POB-Gua) and rapid *O*^6^-alkylguanine-DNA alkyltransferease (AGT)-mediated repair mechanisms (*O*^6^-Me-dGuo). In rats treated with NNK or NNAL for up to 20 or 70 weeks, *O*^2^-POB-Thd or *O*^2^-PHB-Thd clearly predominated among all the quantified DNA adducts. The relatively high levels of *O*^2^-POB/PHB-Thd and its long-term persistence in rat tissues suggest that it might be the mostly likely DNA base adduct to be detected in the tissues of people who use tobacco products. Meanwhile, the spontaneous depurination of *N*7-POB-Gua provides a good chance of detecting this adduct in urine. The specificity of these adducts to tobacco use (as opposed for example to methyl DNA adducts) makes them particularly attractive for mechanistic and biomonitoring studies.

POB and PHB DNA phosphate adducts formed by NNK/NNAL metabolism contribute to a significant portion of the total quantified DNA adducts in rat tissues. The persistence of these phosphate DNA adducts also appears to be longer than many DNA base adducts. However, difficulties in synthesizing the chemical standards of DNA phosphate adducts currently inhibit their accurate quantitation. The lack of apparent mutagenic properties in some POB DNA phosphate adducts (see Section 3.4) also raises some questions regarding their importance in tobacco carcinogenesis.

### 3.4. Mutagenicity and Genotoxicity of POB and PHB DNA Adducts

A recent review by Peterson [116] provided a summary of the genotoxic properties of NNK-derived DNA damage, including the methyl DNA base adducts *O*^6^-Me-dGuo and *N*7-Me-dGuo and minor methyl DNA base adducts, the POB DNA base adducts *O*^6^-POB-dGuo, *O*^2^-POB-Thd and basic sites, as well as aldehyde DNA adducts. PHB DNA base adducts are missing from the adduct panel in the review due to the lack of research data. The studies on the mutagenicity and genotoxicity of DNA phosphate adducts are also limited, with some early studies summarized by Farmer et al. in 2010 [100]. Since then, several studies have provided new insights towards the understanding of the mutagenicity and genotoxicity of some POB and PHB DNA base and phosphate adducts (Table 2).

In *E. coli* cells, *O*^6^-POB-dGuo and *O*^6^-PHB-dGuo impeded DNA replication to a relatively small or moderate extent, respectively. They both primarily induced G-to-A transitions, but *O*^6^-POB-dGuo also induced G-to-T transversions [117]. Similarly, *O*^6^-POB-dGuo produced G-to-A transitions and G-to-T transversions and other, more complex, types of mutation in human kidney cells [118]. Computational calculations revealed that both lesions formed stable pairs with C and T, with the latter pair being the least distorted, which agreed well with the experimental data [122]. The NMR data suggested that *O*^6^-POB-dGuo adopted wobble base pairing with C. An unusual hydrogen bond occurred between the POB carbonyl oxygen and the partner dCyd’s extra amino proton, forming a triplex and twisting the phosphodiester backbone at the lesion site [123]. *O*^6^-PHB-dGuo was suggested to be more mutagenic by stabilizing an intercalated DNA conformation for a deletion mutation [122]. However, no experimental data are currently available to support this computational result. The DNA polymerases induced by SOS were found to play redundant roles in the bypass of two alkylated *O*^6^-dGuo lesions [117]. While *O*^6^-POB-dGuo has been well documented as a substrate of the AGT enzyme [89,124], *O*^6^-PHB-dGuo was also found to be a substrate of this enzyme in one recent study [107].

*O*^2^-POB-Thd is a potent mutagen in SOS-induced *E. coli* and human cells. In SOS-induced wild-type *E. coli*, *O*^2^-POB-Thd induced a mutation frequency of 56%, with the major mutation being T-to-G followed by T-to-A. The T-to-G mutation was reduced in the SOS polymerase-deficient strains [120]. It moderately blocked the replication of DNA and induced almost exclusive T-to-A mutations with a frequency of ~15% in mammalian cells. Multiple translesion synthesis (TLS) DNA polymerases, especially Pol η and Pol ζ, contribute to bypassing *O*^2^-POB-Thd [119,121]. Nucleotide excision repair (NER) has been suggested to be involved in repairing this lesion [94,125]. *O*^2^-PHB-Thd is also repaired by the NER machinery [107].

*O*^4^-POB-Thd was first identified in NNKOAc-treated mammalian cells at levels substantially lower than *O*^2^-POB-Thd [94]. Similarly, *O*^4^-PHB-Thd also occurred at very low levels in the genomic DNA of mammalian cells exposed to NNALOAc compared to *O*^2^-PHB-Thd [107]. Although it is less preferentially formed, *O*^4^-POB-Thd has been found to be mutagenic, exclusively inducing T-to-C transversions at ~35% frequency. Similar to *O*^2^-POB-Thd, Pol η and Pol ζ are the two polymerases that primarily bypass *O*^4^-POB-Thd [119]. The mutagenicity of *O*^4^-PHB-Thd remains uninvestigated. However, it is bypassed by the NER machinery. The AGT enzyme seems to be involved in the repair of this lesion [107].

The mutagenicity and genotoxicity of DNA phosphate adducts have not been extensively investigated. In *E. coli* cells, the *S*_p_ configuration of the methyl DNA phosphate adduct was bypassed with higher efficiency than the *R*_p_ configuration, which showed suppressed DNA replication. The *S*_p_ methyl DNA phosphate adduct induced TT-to-GT and TT-to-GC mutations at the flanking TT dinucleotide site, in an AGT (also termed as Ada)-dependent manner. The replication outcome appeared to be sequence-context-independent of the 5′ dinucleotide adjacent to the lesion. The *R*_p_ diastereomer exhibited an error-free replication bypass. SOS-induced TLS polymerases did not appear to be responsible for bypassing this type of DNA lesion [126,127]. The only genotoxicity study of POB DNA phosphate adducts was conducted in *E. coli* cells by Wu and Wang in 2020 [105]. Surprisingly, no configurations of POB DNA phosphate adducts were found to cause perturbations in DNA replication or mutations in the flanking TT dinucleotides in *E. coli* cells. They were bypassed independently on SOS-induced DNA polymerases and Ada [105].

### 3.5. NNK-Derived DNA Adduct Formation in Lung Carcinogenesis

Deuterium isotope effects were clearly observed in the mutagenicity and carcinogenicity of NNK. In a comparative A/J mouse study, [CD_3_]NNK **90** (Figure 5) induced significantly higher lung tumor multiplicity than NNK, whereas [4,4-D_2_]NNK **91** was considerably weaker at inducing lung tumorigenesis [128]. The levels of *O*^6^-Me-Gua in the pulmonary DNA of mice treated with [4,4-D_2_]NNK were significantly lower than in those treated with NNK or [CD_3_]NNK [128,129]. This was in line with the hypothesis that DNA methylation, particularly *O^6^*-Me-Gua formation in pulmonary cells, is critical in A/J mouse lung carcinogenesis by NNK [28].

Meanwhile, NNKOAc significantly increased lung tumor multiplicity in A/J mice treated with 0.25 or 0.5 μmol acetoxymethylmethylnitrosamine **94** (AMMN, a model methylating agent, Figure 5) [130]. This enhancement was likely due to the depletion of AGT activity by POB DNA adducts [131,132]. The DNA pyridyloxobutylation by NNK prolonged the persistence time of *O*^6^-Me-Gua in mouse lung tissue [79], providing further evidence of the importance of *O*^6^-Me-Gua in A/J mouse lung tumorigenesis.

When F344 rats were treated with [^14^CH_3_]NNK **92** or [carbonyl-^14^C]NNK **93** (Figure 5), a higher incorporation of tissue-bound radioactivity in the lungs was observed from [^14^CH_3_]NNK than from [carbonyl-^14^C]NNK [133]. A comparative carcinogenicity study in rats using [CD_3_]NNK and [4,4-D_2_]NNK indicated that the pyridyloxobutylation of DNA plays an important role in NNK carcinogenesis, at least in the induction of rat nasal-cavity tumors [129,134]. Methyl DNA adducts, such as *O*^6^-Me-Gua and *N*7-Me-Gua, accumulated and persisted in the DNA of rat target organs (lung, nasal cavity, liver) upon NNK treatment [111]. Two types of DNA adduct—the methyl and POB DNA adducts formed by the α-hydroxylation of NNK—are considered to contribute to NNK mutagenicity and carcinogenicity through a synergistic mechanism.

### 3.6. Human DNA Adducts Related to NNK Metabolism

The two types of DNA adduct—HPB-releasing DNA adducts and methyl DNA phosphate adducts—discussed below are not specific to NNK metabolism; they may also result from other sources. NNN metabolism also leads to the formation of HPB-releasing DNA adducts, while some unknown sources in tobacco extracts may also produce these DNA adducts (Ma B, Hecht SS, unpublished data). The formation of methyl DNA phosphate adducts in humans may arise endogenously or from other DNA-methylating sources.

#### 3.6.1. HPB-Releasing DNA Adducts in Human Tissues

As summarized in Table 1, the HPB released upon the acid hydrolysis of the peripheral lung and tracheobronchus DNA of cigarette smokers amounted to 11 ± 16 and 16 ± 18 fmol/mg DNA, respectively, in contrast to the low levels found in the DNA of the same tissues from nonsmokers (0.9 ± 2.3 fmol/mg peripheral lung DNA and 0.9 ± 1.7 fmol/mg tracheobronchus DNA) [80]. In the peripheral lung tissues of patients who had adenocarcinoma or squamous cell carcinoma or another diagnosis (1 patient), a significantly higher level of HPB-releasing DNA adducts was observed in self-reported smokers (404 ± 258 fmol/mg DNA) than in self-reported nonsmokers (59 ± 56 fmol/mg DNA) (*p* < 0.0001) [84]. However, a statistical difference in HPB-releasing adducts between smokers and non-smokers was not observed in the lung DNA of tumor-free sudden-death victims whose smoking status was verified by blood or urinary cotinine levels. The levels of HPB-releasing DNA adducts in the mucosa of the esophagus and cardia from the same two groups did not differ either [85]. In another study with lower esophageal mucosa from patients with or without upper gastrointestinal disorders, there was also no statistical difference in HPB-releasing DNA adduct levels in smokers (4.80 ± 3.57 pmol/mg DNA) versus nonsmokers (2.86 ± 2.44 pmol/mg DNA) after excluding outliers [86].

The levels of HPB released from the oral cell DNA of smokers were 12.0 pmol/mg DNA (mouthwash) and 44.7 pmol/mg DNA (buccal brushings), which were significantly higher than those from samples from nonsmokers (0.23 pmol/mg DNA) [82]. In a larger study with oral samples collected from 65 smokers, 30 of whom had head-and-neck squamous cell carcinoma, the median HPB releasing DNA adduct level was 6.6 times greater in the cancer patients (1.51 pmol/mg DNA) than in the controls (0.23 pmol/mg DNA) [83].

#### 3.6.2. Methyl Phosphate Adducts in Human Lung

In human lung tumor tissues and adjacent normal lung tissues, methyl DNA phosphate adducts were detected in higher levels in the normal lung tissues of 13 current smokers (13 ± 6 adducts/10^9^ nucleotides) than in 17 nonsmokers (8 ± 4 adducts/10^9^ nucleotides). It is important to note that the levels of methyl DNA phosphate adducts remained constant in the lung tissues of untreated rats throughout a 70-week study course, occurring at 5–7 adducts/10^9^ nucleotides. This indicates that the endogenous sources contributing to the formation of methyl DNA phosphate adducts do not accumulate with aging [135].

## 4. Metabolism and DNA Adduct Formation of NNN

### 4.1. Occurrence and Carcinogenicity

NNN is a nonvolatile *N*-nitrosamine present in virtually all tobacco products. During the tobacco curing and aging process, NNN is formed from the nitrosation of nornicotine, a secondary tobacco alkaloid resulting from the demethylation of nicotine via the catalysis of nicotine demethylase [19,136]. NNN can also be formed endogenously through the facile nitrosation of nornicotine, which is present in all tobacco products and is a metabolite of nicotine [137]. NNN, but not NNK, was detected at low levels (~0.5 × 10^−3^% of nicotine dose) in the urine of rats treated with nicotine and sodium nitrite [138]. It was occasionally detected in very low levels in the urine of some users of oral nicotine replacement therapy products [139,140]. NNN has also been detected in e-cigarette users, with median values of 2.15 pg/mL and 0.1 fmol/mL in the saliva and urine, respectively [141].

The first carcinogenicity study of NNN was reported in 1964, in which mice were administered NNN by i.p. injection once weekly. Pulmonary lesions and adenomas, as well as local invasion of the lungs and bronchii were observed during the first 7 months of treatment [142]. Since then, numerous studies with NNN have demonstrated its strong carcinogenicity in laboratory animals, including mice, rats, Syrian golden hamsters and mink [6,20]. NNN exerts its carcinogenicity on target organs, including the nasal cavity, oral cavity, esophagus, lungs and trachea. The organ specificity of NNN carcinogenicity is species- and administration-pathway-dependent [6]. For example, NNN primarily causes lung adenoma in mice after i.p. injection [19,29,143]. It induces tumors of the esophagus, oral cavity and nasal cavity when administered in drinking water [143,144,145,146,147,148], but predominantly causes nasal cavity tumors after s.c. injection in rats [149,150,151]. NNN together with NNK also induced oral cavity tumors through chronic oral swabbing [21]. Due to the chirality of the 2′-carbon, NNN is comprised of two enantiomers. In our chronic rat study of racemic NNN or NNN enantiomers in drinking water for 17–20 months, (*S*)-NNN was the stronger carcinogenic NNN enantiomeric component, causing a high incidence of oral and esophageal tumors at 14 ppm, while (*R*)-NNN was weakly carcinogenic but showed a synergistic enhancement of the carcinogenicity of (*S*)-NNN [148].

### 4.2. Metabolism

**Scheme 5:**
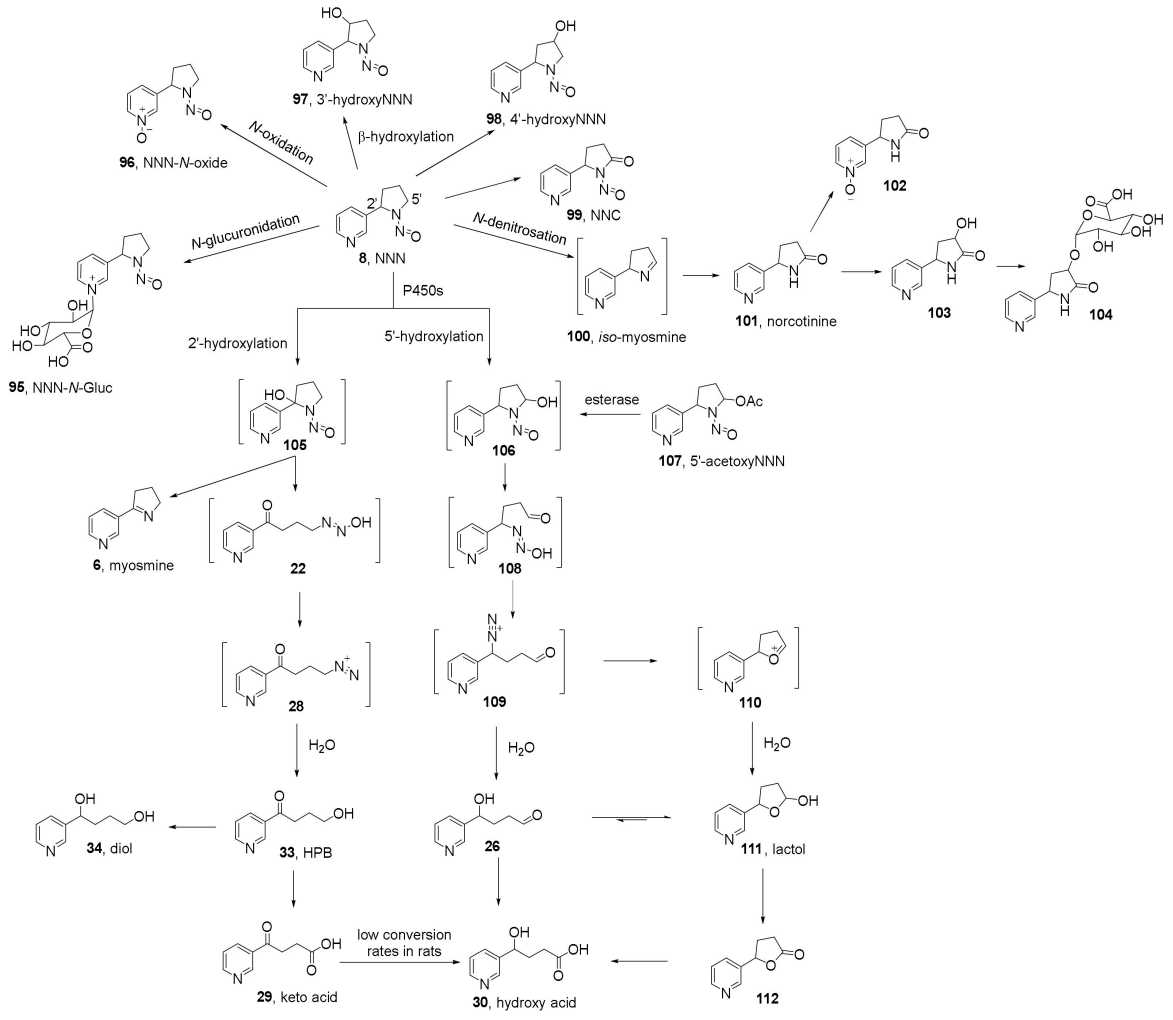
Pathways of NNN metabolism.

To exert its carcinogenicity, NNN requires metabolic activation, primarily catalyzed by P450s 2A6 and 3A4 [6]. The identified metabolic pathways of NNN include (1) α-hydroxylation (2′- and 5′-hydroxylation), (2) β-hydroxylation (3′- and 4′-hydroxylation), (3) pyridine-*N*-glucuronidation, (4) pyridine-*N*-oxidation and (5) denitrosation (Scheme 5). Among the five metabolic pathways, NNN α-hydroxylation is considered to be the major metabolic activation pathway, due to its potential to generate DNA alkylating agents and to form corresponding adducts with DNA or other biomolecules.

#### 4.2.1. NNN α-Hydroxylation

NNN α-hydroxylation consists of two metabolic pathways—2′-hydroxylation and 5′-hydroxylation—due to the asymmetry of its pyrrolidine ring (Scheme 5). Through the catalysis of, primarily, P450 3A4 and, to some extent, 2A4, 2A5 and 2A13 for only (*R*)-NNN [149,152], NNN undergoes 2′-hydroxylation and forms the unstable intermediate 2′-hydroxyNNN **105**, which spontaneously opens and generates a highly reactive electrophile, the POB diazonium ion **28**. 2′-HydroxyNNN also undergoes denitrosation, followed by the loss of one molecule of H_2_O and forms myosmine **6**. The POB diazonium ion **28** forms HPB **33** by solvolysis. The metabolites of HPB are the diol **34** and the keto acid **29**. 

NNN 5′-hydroxylation is mainly catalyzed by P450 2A6 but also, to a lesser extent, by P450s 2E1 and 2D6 [149,152,153]. After oxidation, the unstable product 5′-hydroxyNNN **106** forms the diazohydroxide **108**, which spontaneously loses one molecule of H_2_O and forms the 4-oxo-1-(pyridine-3-yl)butyl (OPB) diazonium ion **109**. The OPB diazonium ion can also rearrange to form the oxonium ion **110**. The two ions, **109** and **110**, are both highly electrophilic and react with DNA to form adducts. They also undergo solvolysis reactions and form lactol **111** and its ring-opened form **26** in equilibration, both of which are further metabolized to the hydroxy acid **30**.

(1) Overall Metabolic Profiles of NNN in Laboratory Animals

The first study of NNN metabolism was conducted by us in 1978 [150]. Using [2′-^14^C]NNN, 73–85% of the total radioactivity of the dosed compound was detected in the 48-h urine of F344 rats administered NNN by a single s.c. injection. The major metabolites of NNN in rat urine were identified as keto acid **29** and hydroxy acid **30** [150]. The urinary metabolite profiles were qualitatively similar in F344 rats, strain-A mice and Syrian golden hamsters, regardless of the administration pathways. Hydroxy acid **29** (37–53%, respectively, of the dose), keto acid **30** (8–31%), NNN-*N*-oxide **96** (7–11%), NNN **8** (3–5%) and norcotinine **101** (3–5%) were the principal urinary metabolites [58,154,155]. NNN metabolites formed in patas monkeys were investigated after i.v. injection with [5-^3^H]NNN [151]. Hydroxy acid **29** was the principal metabolite, accounting for 43.8 ± 4.0% of the total radioactivity eluting from the HPLC. The other metabolites were 3′-hydroxynorcotinine **103** (16.9%), norcotinine-1*N*-oxide **102** (16.5%), norcotinine **101** (13.1%), 3′-(*O*-β-D-glucopyranuronosyl)hydroxynorcotinine **104** (5.4%) and keto acid **29** (2.7%). Unchanged NNN accounted for only 0.63 ± 0.15%. Hydroxy acid **29** and norcotinine **101** were the two major metabolites in monkey serum [151]. 3′-Hydroxynorcotinine **103** was quantified in human urine at levels of 393 ± 287 pmol/mL in smokers and 658 ± 491 pmol/mL in current nicotine patch users who were former smokers. This metabolite was not detected in any nonsmoker urine. Considering its relatively high levels in urine samples, it is likely that the detected 3′-hydroxynorcotinine was formed as a minor metabolite of nicotine [156].

(2) Preferential α-Hydroxylation of NNN in Target Tissues

Differential tissue metabolism might provide some insights in determining the organospecific carcinogenicity of *N*-nitrosamines. Compared to NNK, NNN administered in drinking water was carcinogenic to the oral mucosa and esophagus of rats [143,144,145,146,147,148]. This was partially explained by the higher activity of the esophagus in metabolizing NNN than NNK [157]. The organospecificity of NNN, causing esophageal tumors compared to hepatic tumors, may also be due to the presence of a high-affinity microsomal enzyme in the rat esophagus, which can efficiently catalyze the α-hydroxylation of *N*-nitrosamines, including NNN [154,155,158,159]. The apparent *K*_M_ for the total α-hydroxylation of NNN by rat esophageal microsomes was much lower (49 ± 6.5 μM) than that by rat liver microsomes (1.2 ± 0.25 mM) [159].

(3) Selectivity of α-Hydroxylation of NNN in Target Tissues

Keto acid **29** and hydroxy acid **30** are the end metabolites resulting from 2′- and 5′-hydroxylation of NNN in F344 rats, respectively. They were not formed from norcotinine **101**, and no significant interconversion (1%) between those two metabolites was observed based on analysis of rat urine [60,160]. Thus, keto acid and hydroxy acid can serve as reliable biomarkers for assessing the two α-hydroxylation pathways of NNN metabolism in laboratory animal models. By quantifying the concentrations of **29** and **30** in in vitro incubation mixtures and in rat urine, catalytic rates of α-hydroxylation of NNN at 2′- and 5′-carbons can be established [161]. However, this does not hold true in humans. A high conversion rate (85%) of keto acid to hydroxy acid was observed based on analysis of human urine [59].

Preferential hydroxylation of NNN at the 2′-carbon has been consistently observed in rat esophagus. In cultured rat esophagus, the ratio of 2′-/5′-hydroxyation metabolites reached 3.4 in a 24 h incubation [157,162]; in rat esophageal microsomes, the ratio was 3.2 ± 0.5 at various NNN concentrations (from 1 μM to 2 mM) [159]. Similarly, 2′-hydroxylation predominated in rat nasal mucosa and rat oral tissues [157,163]. However, it is interesting that 5′-hydroxylation is preferentially observed in rat liver. While both hydroxylation pathways by rat liver microsomes have a similar *K*_M_ of ~2 mM, the *V*_max_ of 5′-hydroxylation (1.05 nmol/min/mg protein) is nearly 2-fold higher than that of 2′-hydroxyaltion (0.53 nmol/min/mg protein) [161]. The ratio of 2′-/5′-hydroxyation metabolites in rat liver microsomes was 0.71–0.23 depending on NNN concentration [159]. 5′-Hydroxylation also predominated in A/J mouse lung (ratio of 2′-/5′-hydroxyation metabolites: 0.6) and hamster esophagus and trachea (ratio of 2′-/5′-hydroxyation metabolites: 0.3 and 0.7, respectively) [29,164] and in some human tissues including buccal mucosa, trachea, esophagus, lung and bladder [30]. In patas monkeys, hydroxy acid significantly exceeded keto acid in serum concentrations, indicating preferential metabolism by the 5′-hydroxylation pathway in this species [151].

The interspecies and interindividual differences in the selectivity of NNN α-hydroxylation are remarkable. Liver microsomes from Aroclor-pretreated F344 rats showed a 20-fold induction of 2′-hydroxylation of NNN but only a 1.9-fold induction of NNN 5′-hydroxylation. The *V*_max_’s for NNN 2′- and 5′-hydroxylation by the treated rat liver microsomes were 0.53 and 1.05 nmol/min/mg protein, respectively [161]. The ability to catalyze NNN 2′- and 5′-hydroxylation by human liver microsomes was lower, with mean rates of 0.04 and 0.05 nmol/min/mg protein respectively [165]. A high interindividual variation of the ability of human tissues to metabolize NNN has been observed. Keto acid **29** was not detectable in most individuals but high in one case, similar to a 75-fold variation of benzo[*α*]pyrene metabolism in cultured human bronchi [30,166].

#### 4.2.2. NNN β-Hydroxylation

NNN β-hydroxylation generates two products—3′-hydroxyNNN **97** and 4′-hydroxyNNN **98** (Scheme 5). They have been detected in the urine of rats administered NNN, however, only at trace levels. The rates of formation of both metabolites by rat liver microsomes were less than 0.01 nmol/min/mg protein [167].

#### 4.2.3. NNN Detoxification Pathways

Pyridine-*N*-oxidation and glucuronidation of NNN are considered to be the two major detoxification pathways of NNN metabolism. Denitrosation products have also been observed in vivo to a moderate extent.

The metabolite formed by pyridine-*N*-oxidation is *N*′-nitrosonornicotine-1*N*-oxide **96** (NNN-*N*-oxide, Scheme 5). It accounted for 6.7–9.4% or 10.8% of the dose of NNN in the urine of rats treated by s.c. injection or by gavage, respectively [167,168]. Comparative studies in F344 rats and Syrian golden hamsters demonstrated that NNN-*N*-oxide was less carcinogenic than NNN, indicating that pyridine-*N*-oxidation is a detoxification pathway of NNN metabolism [143]. This metabolite has been observed in incubation mixtures of human liver microsomes and cultured human tissues [30,156,169,170,171].

NNN glucuronidation, likely catalyzed mainly by UGT1A4 [172], generates NNN pyridine-*N*-glucuronide **95** (NNN-*N*-Gluc, Scheme 5). It has been identified in human urine, accounting for 59.1 ± 26.0% and 61.8 ± 7.7% of the total of free plus glucuronidated NNN in smokers and smokeless tobacco users, respectively [173]. Urinary NNN-*N*-Gluc together with urinary free NNN comprise “total NNN”, which has successfully served as a biomarker for assessing esophageal cancer risk in smokers [169].

Through denitrosation and sequential oxidation reactions, NNN forms norcotinine **101** and its downstream metabolites likely via the intermediate *iso*-myosmine **100** [151]. However, mechanisms proceeding via nornicotine or 5′-hydroxyNNN **106** could not be excluded [6]. Norcotinine **101** has been detected in vitro in an incubation mixture of NNN with cultured mouse lung [29] and in vivo in rat urine [160,174]. It accounted for 3–5% of total urinary metabolites of NNN in rats, mice and hamsters [58,154,155] and 29.6% (plus its *N*-oxidation metabolite) of total NNN metabolites in the serum of patas monkeys [151].

#### 4.2.4. Other Possible Metabolic Pathways

Mediated by P450 2A6, NNN is oxidized to form the nitrosamide *N*′-nitrosonorcotinine **99** (NNC, Scheme 5) as a minor metabolite in vitro. This nitrosamide product has an in vitro half-life of 12.3 min [54].

#### 4.2.5. Stereochemistry of NNN Metabolism

(*S*)-NNN and (*R*)-NNN are the two enantiomers of NNN existing in tobacco and tobacco products, with (*S*)-NNN predominating in tobacco [170,175]. Our chronic carcinogenicity study has clearly shown that (*S*)-NNN is a strong oral and esophageal carcinogen in rats whereas (*R*)-NNN was less carcinogenic but a potent co-carcinogen to (*S*)-NNN [148]. The potential difference of metabolic activation of NNN enantiomers in target tissues may be responsible for the observed differences in carcinogenicity.

For NNN α-hydroxylation, enantiomeric differences have been observed in vitro with P450 2As [152]. The kinetic parameter *K*_M_ for (*R*)-NNN 5′-hydroxylation is 22 ± 6 μM, which is significantly higher than that of (*S*)-NNN (2.3 ± 0.6 μM). For 2′-hydroxylation, all P450 2As except 2A6 metabolized (*R*)-NNN but none were active for (*S*)-NNN [152]. In cultured rat esophagus, (*S*)-NNN was preferentially metabolized to 2′-hydroxylation products, whereas (*R*)-NNN was predominantly metabolized by 5′-hydroxylation. This observation also held true in vivo in rats treated with (*R*)- or (*S*)- or racemic NNN by gavage [171].

Due to the potential difference of in vivo metabolism of the two NNN enantiomers, the enantiomeric composition of NNN in human urine may be different from that in tobacco products. In 2000, we analyzed the enantiomers of NNN in commercial cigarette tobacco and smokeless tobacco products—moist snuff and chewing tobacco—in the U.S. market. (*S*)-NNN comprised 75 ± 8.8% of total NNN in 12 products [175]. An updated analysis of 37 products in 2012 suggested that (*S*)-NNN was still the predominant enantiomer, representing for 62.9 ± 6.3% of total NNN [170]. Using a chiral stationary phase mass spectrometry method, we quantified (*S*)- and (*R*)-NNN in the urine of smokers and smokeless tobacco users; (*S*)-NNN comprised 67 ± 5% (*N* = 20) and 56 ± 3% (*N* = 10) of total NNN respectively [176]. Considering the remarkable similarity of the enantiomeric compositions of NNN in tobacco products and human urine, the overall metabolic biotransformation of (*S*)-NNN and (*R*)-NNN probably does not differ much in humans.

### 4.3. DNA Adducts Formed by NNN Metabolism

As depicted in Scheme 5, intermediates formed by NNN 2′-hydroxylation such as the POB diazonium ion **28** and by NNN 5′-hydroxylation such as the OPB diazonium ion **109** and the oxonium ion **110** have the electrophilic power to react with DNA and form the corresponding DNA adducts. While POB DNA adducts formed by NNN 2′-hydroxylation are the same as NNK-derived adducts, adducts formed by NNN 5′-hydroxylation are structurally unique. They represent a group of DNA adducts with the potential to be NNN-specific bioactivation biomarkers to monitor chronic exposure, uptake, and metabolic activation of this very important tobacco-specific carcinogen.

#### 4.3.1. DNA Adducts Formed by NNN 2′-Hydroxylation

Due to the convergence of the formation of the POB diazonium ion **28** by NNN 2′-hydroxylation (as shown in Scheme 5) and NNK α-methyl hydroxylation (as shown in Scheme 2), most of the POB DNA base and phosphate adducts formed by NNN are similar to those formed by NNK in vitro and in vivo.

(1) POB DNA Base Adducts Formed by NNN 2′-Hydroxylation

The formation of HPB-releasing DNA adducts were readily detected in the liver, lung and nasal mucosa DNA of NNN-exposed mice and rats (Table 1) [75,79,80,81]. The failure to detect HPB-releasing DNA adducts in other tissues was probably due to the detection limit of the analytical method used in those studies [75].

Individual DNA adducts caused by NNN were first characterized and quantified by us in 2007 [177,178]. Three major POB DNA base adducts *O*^2^-POB-Thd **60**, *N*7-POB-Gua **59** and *O*^2^-POB-Cyt **49** (Figure 1) were detected in the target tissues (esophagus, nasal cavity, oral cavity) and livers and lungs of rats treated with 10 ppm (*S*)-NNN or (*R*)-NNN in the drinking water for up to 20 weeks. *O*^6^-POB-dGuo **56** was only occasionally detectable in rat nasal mucosa and oral mucosa, mostly in the (*R*)-NNN treatment group at early time points (weeks 1, 2, 5). At various time intervals, *O*^2^-POB-Thd predominated among all the 4 POB DNA adducts, by exceeding *N*7-POB-Gua in nearly all the examined tissues except for esophagus from both treatment groups. *O*^2^-POB-Cyt was formed at lower levels than *O*^2^-POB-Thd and *N*7-POB-Gua in all tissues except for esophagus from the (*S*)-NNN treatment group at some time points [177,178]. The enantiomeric distribution pattern of those POB DNA adducts formed by NNN is intriguing (Table S2). In the esophagus, oral mucosa and liver, (*S*)-NNN caused higher concentrations of all the POB DNA base adducts than its (*R*)-counterpart; however, the trend was inverted in most cases in the nasal olfactory and respiratory mucosa. This was considered to be due to the hepatic first-pass effect of preferential metabolism of (*S*)-NNN in the liver thus allowing more (*R*)-NNN to circulate to the nasal cavity and form DNA adducts. NNN-derived POB DNA adducts formed in the highest concentrations in the nasal respiratory mucosa, especially after the treatment with (*R*)-NNN [177,178].

**Figure 6:**
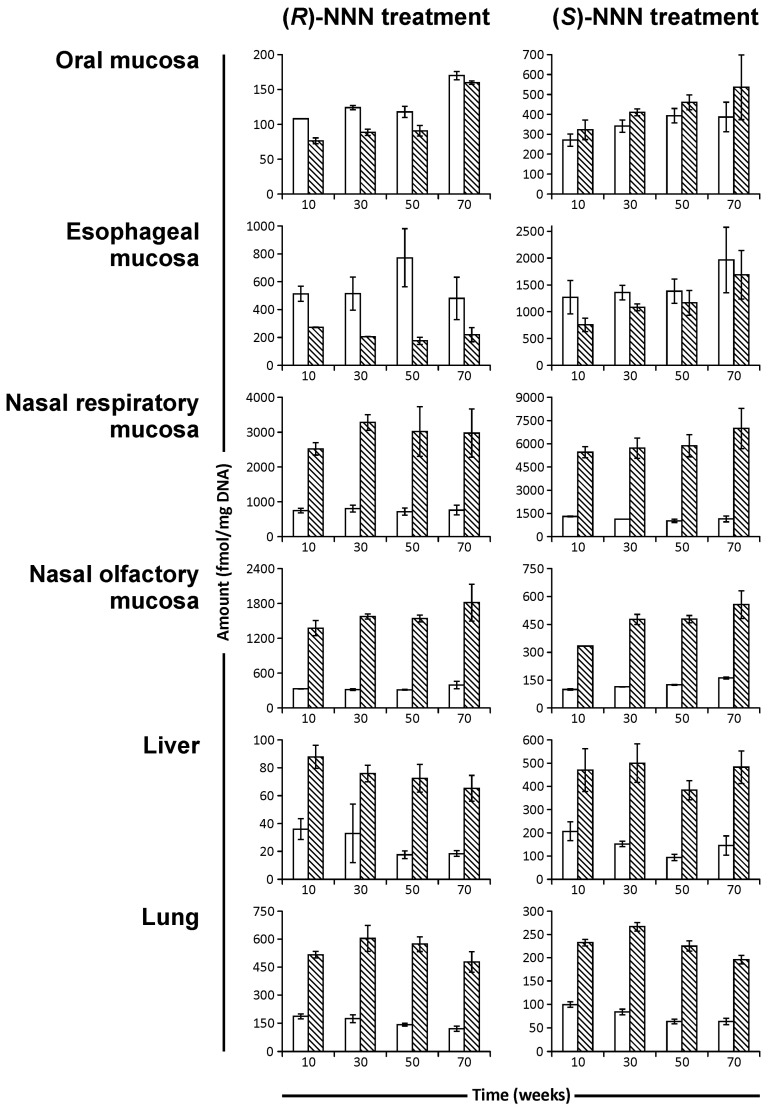
Levels of *N*7-POB-Gua (open bars) and *O*^2^-POB-Thd (striped bars) in the tissues of rats treated with (S)- or (R)-NNN in the drinking water for up to 70 weeks. Adapted with permission from ref. [179]. Copyright 2013 American Chemical Society.

To further understand the persistence and accumulation of NNN-derived POB DNA adducts in rats, we conducted a chronic carcinogenicity study in F344 rats treated with racemic NNN or its enantiomers in the drinking water for up to 17 months [148,179]. Tissues from rats sacrificed at weeks 10, 30, 50 and 70 were collected and analyzed for the levels of *N*7-POB-Gua and *O*^2^-POB-Thd, the two most abundant POB adducts formed by NNN (Figure 6 and Table S2). As shown in Figure 6, *N*7-POB-Gua and *O*^2^-POB-Thd clearly accumulated to the highest levels in the nasal respiratory mucosa of rats in both the (*S*)-NNN and (*R*)-NNN treatment groups. They also persisted in considerably high levels in the nasal olfactory mucosa of rats treated with (*R*)-NNN. *O*^2^-POB-Thd predominated in all the nasal mucosa DNA. However, the nasal mucosa was not the target tissue for carcinogenicity even though the high levels of DNA adducts were formed at this site. This contradiction was likely due to a more deleterious effect on rat survival of the other tumors observed in this study including esophageal and oral tumors than nasal tumors. The relative levels of *N*7-POB-Gua and *O*^2^-POB-Thd in the oral mucosa and esophagus, the two major target tissues for carcinogenicity, were fully consistent with the stronger carcinogenicity of (*S*)-NNN than (*R*)-NNN. The formation of *O*^2^-POB-dCyd (detected as *O*^2^-POB-Cyt) was not observed in the rat tissues from this study [179].

To provide a possible explanation for (*R*)-NNN as a synergistic co-carcinogen to (*S*)-NNN, levels of *O*^6^-POB-dGuo were quantified in esophageal mucosa DNA of rats treated with NNN enantiomers or racemate (Table S2) [180]. The adduct levels were synergistically increased, in a modest but statistically significant extent, in the racemic NNN treatment group compared to the sum of the amounts formed in the (*S*)-NNN group or the (*R*)-NNN group. No synergy in the formation of this adduct was observed in oral cavity or nasal cavity. The formation of other POB DNA base adducts of NNN similarly did not elicit any synergistic enhancement by the treatment of (*R*)-NNN in the racemic NNN group [180].

(2) POB DNA Phosphate Adducts Formed by NNN 2′-Hydroxylation

POB DNA phosphate adducts formed by NNN 2′-hydroxylation were also quantified in the DNA of rats treated with (*S*)-NNN or (*R*)-NNN from our carcinogenicity study (Table S2) [87,148]. The highest amounts of total POB DNA phosphate adducts were observed in the esophageal DNA of rats treated with (*S*)-NNN, peaking at week 50. Levels of POB DNA phosphate adducts caused by (*S*)-NNN in esophagus, oral mucosa and liver were higher than those from (*R*)-NNN treatment. The reverse result was observed in nasal mucosa DNA, which was consistent with our previous results, likely reflecting the hepatic first-pass effect. Some combinations such as Ap(POB)C **64** or Cp(POB)A **65** (Figure 1) persisted throughout the study course of 70 weeks, suggesting their potential as biomarkers for monitoring chronic exposure to NNN and NNK in humans [87].

#### 4.3.2. DNA Adducts Formed by NNN 5′-Hydroxylation

The 5′-hydroxylation pathway generates two unique electrophilic intermediates **109** and **110** (Scheme 5) that are considered to be NNN-specific. Thus, DNA adducts formed by NNN 5′-hydroxylation have the potential to be NNN-specific activation biomarkers for tobacco-related cancer etiology studies.

(1) DNA Base Adducts Formed by NNN 5′-Hydroxylation

**Figure 7:**
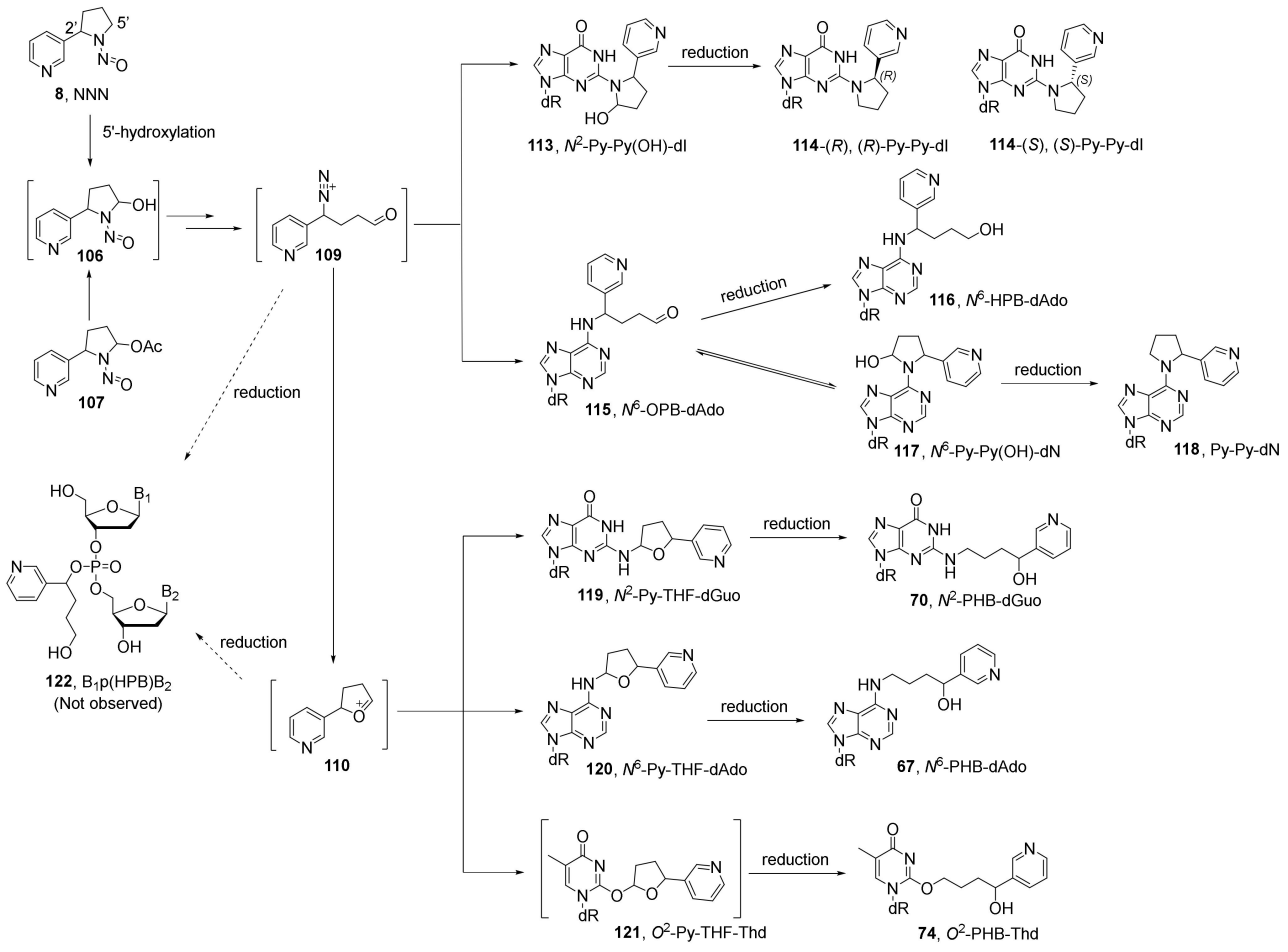
DNA adducts formed by NNN 5′-hydroxylation.

The regiochemically activated precursor of 5′-hydroxyNNN, 5′-acetoxy-*N*′-nitrosonornicotine **107** (5′-acetoxyNNN, Figure 7), was used to investigate DNA adducts possibly formed by the 5′-hydroxylation pathway of NNN metabolism. In the reaction of 5′-acetoxyNNN with dGuo, adducts resulting from NNN 5′-hydroxylation were identified as *N*^2^-[2-(3-pyridyl)-*N*-pyrrolidinyl-5-hydroxy]-2′-deoxyinosine (**113**, *N*^2^-Py-Py(OH)-dI) and *N*^2^-[5-(3-pyridyl)tetrahydrofuran-2-yl]-2′-deoxyguanosine (**119**, *N*^2^-Py-THF-dGuo). Their corresponding reduced forms 2-[2-(3-pyridyl)-*N*-pyrrolidinyl]-2′-deoxyinosine (**114**, Py-Py-dI) and *N*^2^-PHB-dGuo **70** were verified by each authentic synthesized standard. The two reduced adducts **114** and **70** were also detected in the reaction of 5′-acetoxyNNN with calf thymus DNA [181]. Py-Py-dI was also formed in the DNA of rats treated with racemic NNN for 3 weeks [182]. As shown in Table S2, Py-Py-dI preferentially formed in rat lung and nasal mucosa DNA in a dose-dependent manner upon NNN exposure. For example, in the 500 ppm NNN treatment group, Py-Py-dI occurred at 2018 ± 200, 2458 ± 190 and 2015 ± 80 fmol/μmol dGuo in rat lung, nasal olfactory mucosa and nasal respiratory mucosa, respectively. The levels of Py-Py-dI in the other tissues (liver, esophagus and oral mucosa) were substantially lower [182]. The distribution pattern of Py-Py-dI in NNN-treated rats reflected the expression profile of CYP2A3, the primary P450 enzyme responsible for NNN 5′-hydroxylation in rats [152]. It is also interesting to note that the formation of Py-Py-dI is stereospecific. (*S*)-NNN yielded exclusively the (*R*)-enantiomer of Py-Py-dI (**114**-(*R*), Figure 7), whereas (*R*)-NNN yielded nearly equal amounts of the two enantiomers of Py-Py-dI. This probably results from racemization of the OPB diazonium ion **109** formed by (*R*)-NNN [182].

In the reaction of 5′-acetoxyNNN with dAdo, *N*^6^-[5-(3-pyridyl)tetrahydrofuran-2-yl)-2′-deoxyadenosine (**120**, *N*^6^-Py-THF-dAdo) and its reduced form upon NaBH_3_CN treatment—*N*^6^-PHB-dAdo **67**—were observed [183]. They were also detected in calf thymus DNA treated with 5′-acetoxyNNN, but not in rat liver and lung DNA upon treatment with NNN [184]. *N*^6^-[2-(3-Pyridyl)-*N*-pyrrolidinyl-5-hydroxy]-2′-deoxynebularine (**117**, *N*^6^-Py-Py(OH)-dN), on the other hand, has been detected both in vitro and in vivo. It was readily observed in liver and lung DNA of rats treated with 500 ppm racemic NNN in the drinking water for 3 weeks. Its reduced form 6-[2-(3-pyridyl)-*N*-pyrrolidinyl]-2′-deoxynebularine (**118**, Py-Py-dN) was observed only in NaBH_3_CN-reduced rat DNA but not in NaBH_4_-reduced samples. This is likely due to the equilibrium between *N*^6^-Py-Py(OH)-dN **117** and *N*^6^-[4-oxo-1-(pyridine-3-yl)butyl]-2′-deoxyadenosine (**115**, *N*^6^-OPB-dAdo), and the competing reduction of *N*^6^-OPB-dAdo **115** to *N*^6^-[4-hydroxy-1-(pyridine-3-yl)butyl]-2′-deoxyadenosine (**116**, *N*^6^-HPB-dAdo) [184,185]. *N*^6^-HPB-dAdo **116**, structurally specific to NNN metabolism, has been quantified in the liver and lung DNA of rats treated with NNN. Its levels, however, were substantially lower than Py-Py-dI **114** in the same tissues (Table S2) [185].

The reduced adduct *O*^2^-PHB-Thd **74** was also identified in a hydrolysate of 5′-acetoxyNNN-treated calf thymus DNA after NaBH_3_CN reduction. This product was hypothesized to arise from the reduction of the unstable intermediate *O*^2^-[5-(3-pyridyl)tetrahydrofuran-2-yl]thymidine (**121**, *O*^2^-Py-THF-Thd) [183].

(2) DNA Phosphate Adducts Formed by NNN 5′-Hydroxylation

By analogy to our previous studies with POB DNA phosphate adducts formed by NNN 2′-hydroxyation, we hypothesized that DNA phosphate adducts resulting from the OPB diazonium ion **109** and/or the oxonium ion **110** could also be formed in the reaction of 5′-acetoxyNNN with calf thymus DNA or synthesized dinucleotides. Upon reduction, the simplest DNA phosphate adducts were proposed to be B_1_p(HPB)B_2_ **122** (Figure 7). However, no evidence of the formation of such adducts was observed (Li Y. and Hecht S.S. unpublished data).

### 4.4. Mutagenicity and Genotoxicity of NNN-Specific DNA Adducts

**Figure 8:**
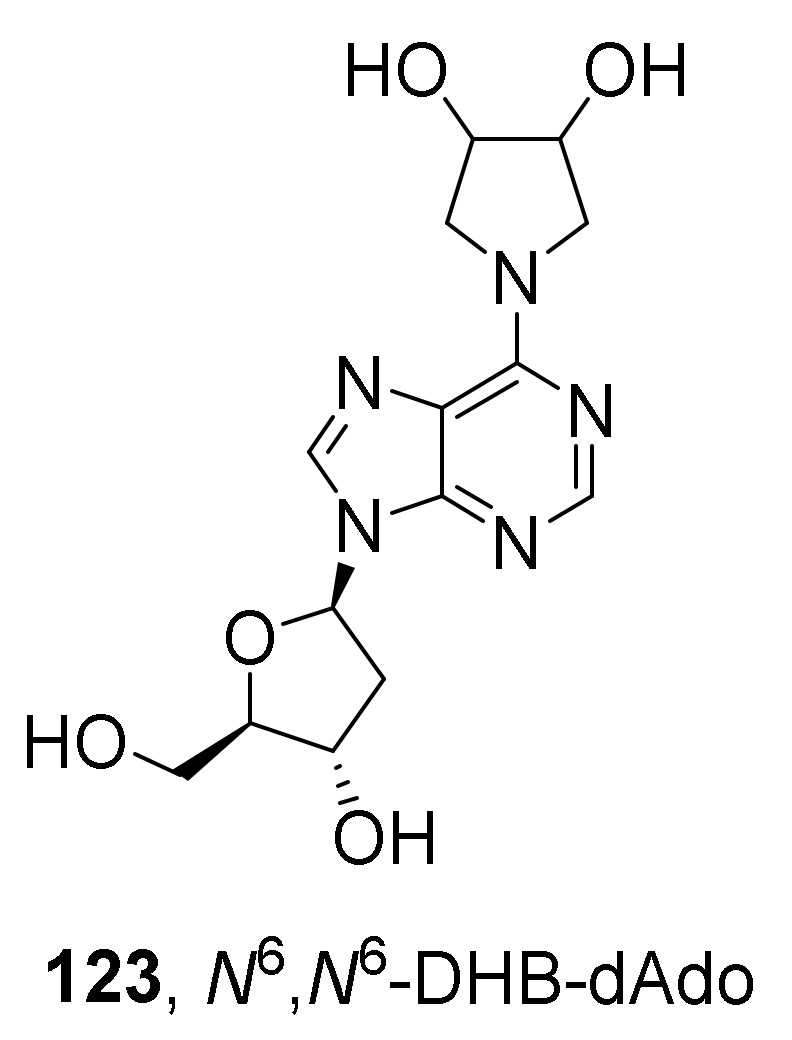
Structure of *N*^6^,*N*^6^-DHB-dAdo.

Most of the reported mutagenicity and genotoxicity studies reported to date have focused on POB DNA base adducts which can be formed from either NNN (by 2′-hydroxylation) or NNK (see the discussion in Section 3.4). There have been no reports on the newly identified NNN-specific DNA base adducts arising from the 5′-hydroxyaltion pathway. However, considering the structural similarity of the DNA adducts formed by NNN 5′-hydroxylation, for example, Py-Py-dI **114** and Py-Py-dN **118**, to other reported mutagenic DNA adducts such as *N*^6^,*N*^6^-(2,3-dihydroxybutan-1,4-diyl)-2′-deoxyadenosine **123** (*N*^6^,*N*^6^-DHB-dAdo, Figure 8), they are reasonably anticipated to be mutagenic and/or genotoxic [186]. Further studies are warranted to elucidate their roles in the carcinogenesis by NNN.

## 5. Concluding Remarks

*N*-nitrosamines comprise a large group of approximately 200 compounds that have been documented to be carcinogenic in more than 30 animal species [187]. Human exposure to these compounds is low in general with some exceptions such as NDMA exposure from certain types of foods or its possible endogenous formation in the stomach [188]. However, exposure to some *N*-nitrosamines such as NNN and NNK among tobacco users occur in far greater amounts. Considering their common occurrence in tobacco products and very strong carcinogenicity in laboratory animals, NNN and NNK are the 2 *N*-nitrosamines that have received the widest attention with regard to their metabolism and DNA interactions as related to tobacco carcinogenesis.

The potent pulmonary carcinogenicity and tobacco-specificity of NNK and its metabolism to NNAL which persists in human urine weeks after NNK exposure ceases [189,190], led to studies on NNAL in the urine of non-smokers exposed to secondhand tobacco smoke. These studies contributed significantly to the scientific foundation for the relationship between secondhand tobacco smoke exposure and lung cancer leading to the current nearly universal smoke free indoor environments which were uncommon when this research began in 1992 [47,191,192,193]. Urinary NNAL measurements in the Population Assessment of Tobacco and Health (PATH) study, involving approximately 46,000 adults in the U.S., demonstrated the highest levels of NNAL in smokeless tobacco users, followed by cigarette smokers, both amounts being far greater than in non-users of any tobacco product [194]. Similar results were obtained in the U.S. National Health and Nutrition Examination Survey (NHANES) [195]. Urinary NNAL levels in cigarette smokers were significantly related to lung cancer risk in the prospective Shanghai Cohort Study and Singapore Chinese Health Study, both of which collected the samples years before lung cancer developed [196,197]. These data collectively provide enhanced regulatory rationales related to tobacco use.

The metabolic pathways and DNA adduct structures presented here provide the basis for new insights on mechanisms of *N*-nitrosamine carcinogenesis. These data can lead to the development of biomarkers of carcinogen exposure and cancer risk. This is particularly critical in exposure scenarios such as smokeless tobacco use in South-East Asia where the relatively high levels of NNN in some products are plausible causes for the commonly occurring oral cancers in that region of the world.

DNA adduct formation caused by *N*-nitrosamines is considered the necessary step leading to tumor formation [9]. It is important to investigate DNA adducts that are structurally specific to the exposed carcinogen, and quantify the levels of such DNA lesions in human tissues so that predictive or diagnostic information can be obtained relating to cancer incidence in the affected population. While great progress has been made over the past decades, many unknowns remain. For example, the organospecificity of NNK to lung and of NNN to oral, nasal cavities and esophagus has been very well documented by numerous laboratory and epidemiology studies. However, no NNN/NNK-specific DNA adduct (such as POB- and PHB-DNA adducts discussed above) has been detected in the tissues of human tobacco users except for the structurally uncharacterized HPB-releasing DNA adduct. Difficulties in obtaining relevant tissue samples, the timing with respect to exposure, and the low amounts of these carcinogens (a few micrograms per day) taken up even in regular cigarette smokers, are among the principal reasons. Further studies are required on this critical topic.

In summary, much progress has been made in understanding the metabolism and DNA interactions of the tobacco-specific carcinogenic *N*-nitrosamines NNN and NNK over the past 2–3 decades. We herein provide a comprehensive and updated review on these topics hoping to facilitate research relevant to cancer etiology and prevention.

## Figures and Tables

**Table 2:** Mutagenicity of some POB and PHB DNA adducts.

DNA Adduct	Mutation	Cell	Ref.
POB DNA adduct			
*O*^6^-POB-dGuo	G > A (~90%) and G > T (~2%)	*E. coli*	[117]
	G > A (90%); G > T (~3%); other more complex types	HEK293	[118]
	G > A (~75%) and G > T (~3%)	HEK293T	[119]
*O*^2^-POB-Thd	T > G (37%) and T > A (12%)	*E. coli*	[120]
	T > A (47%)	HEK293T	[121]
	T > A (~15%)	HEK293T	[119]
*O*^4^-POB-Thd	T > C (~35%)	HEK293T	[119]
B_1_p(POB)B_2_	Not mutagenic	*E. coli*	[105]
PHB DNA adduct			
*O*^6^-PHB-dGuo	G > A (~95%)	*E. coli*	[117]

## Supplementary Materials

The following supporting information can be downloaded at: https://www.mdpi.com/article/10.3390/ijms23095109/s1.

## Abbreviations

5′-AcetoxyNNN	5′-acetoxy-*N*′-nitrosonornicotine
AGT	*O*^6^-alkylguanine-DNA alkyltransferease
AKRs	aldo-keto reductases
AMMN	acetoxymethylmethylnitrosamine
dIno	2′-deoxyinosine
dUrd	2′-deoxyuridine
*E. Coli*	*Escherichia coli*
HPB	4-hydroxy-1-(3-pyridyl)-1-butanone
11β-HSD1	11β-hydroxysteroid dehydrogenase type 1
IARC	International Agency for Research on Cancer
i.p.	intraperitoneal
i.v.	intravenous
*N*^2^-POB_b_-dGuo	*N*^2^-(4-(3-pyridyl)-4-oxobut-2-yl)-2′-deoxyguanosine
Py-Py-dI	2-[2-(3-pyridyl)-*N*-pyrrolidinyl]-2′-deoxyinosine
Py-Py-dN	6-[2-(3-pyridyl)-*N*-pyrrolidinyl]-2′-deoxynebularine
*N*^2^-Py-Py(OH)-dI	*N*^2^-[2-(3-pyridyl)-*N*-pyrrolidinyl-5-hydroxy]-2′-deoxyinosine
*N*^6^-Py-Py(OH)-dN	*N*^6^-[2-(3-Pyridyl)-*N*-pyrrolidinyl-5-hydroxy]-2′-deoxynebularine
*N*^2^-Py-THF-dGuo	*N*^2^-[5-(3-pyridyl)tetrahydrofuran-2-yl]-2′-deoxyguanosine
*N*^6^-HPB-dAdo	*N*^6^-[4-hydroxy-1-(pyridine-3-yl)butyl]-2′-deoxyadenosine
*N*^6^-OPB-dAdo	*N*^6^-[4-oxo-1-(pyridine-3-yl)butyl]-2′-deoxyadenosine
*N*^6^,*N*^6^-DHB-dAdo	*N*^6^,*N*^6^-(2,3-dihydroxybutan-1,4-diyl)-2′-deoxyadenosine
NAB	*N*′-nitrosoanabasine
NADP	nicotinamide adenosine dinucleotide phosphate
NAT	*N*′-nitrosoanatabine
NER	nucleotide excision repair
NHANES	National Health and Nutrition Examination Survey
NNAL	4-(methylnitrosamino)-1-(3-pyridyl)-1-butanol
NNALOAc	4-(acetoxymethylnitrosamino)-1-(3-pyridyl)-1-butanol
NNAL-*N*-Gluc	4-(methylnitrosamino)-1-(3-pyridyl-*N*-β-D-glycopyranuronosyl)-1-butanolonium inner salt
NNAL-*O*-Gluc	4-(methylnitrosamino)-1-(3-pyridyl)-1-(*O*-β-D-glucopyranuronosyl)butane
NNC	nitrosamide *N*′-nitrosonorcotinine
NNK	4-(methylnitrosamino)-1-(3-pyridyl)-1-butanone
NNKOAc	4-((acetoxymethyl)nitrosamino)-1-(3-pyridyl)-1-butanone
NNN	*N*′-nitrosonornicotine
OPB	4-oxo-4-(pyridin-3-yl)butanal
OPB diazonium ion	4-oxo-1-(pyridine-3-yl)butyl diazonium ion
PATH study	Population Assessment of Tobacco and Health study
PHB	pyridylhydroxybutyl
POB	pyridyloxobutyl
pol κ	polymerase κ
ppm	parts per million
s.c.	subcutaneous
*S. typhinurium*	*Salmonella typhinurium*
TLS	translesion synthesis
TSNAs	tobacco-specific *N*-nitrosamines
