# Supplementary material for: Metabolism and DNA Adduct Formation of Tobacco-Specific N-Nitrosamines
Source: Int J Mol Sci. 2022 May 4;23(9):5109. doi: 10.3390/ijms23095109 (PMC9104174; doi:10.3390/ijms23095109)
Supplement: Supplementary file 1 [file ijms-23-05109-s001.zip › ijms-1680993-supplementary.pdf]

Supplementary Information

Metabolism and DNA Adduct Formation of Tobacco-specific *N*-Nitrosamines

Yupeng Li [a,b,*] and Stephen S. Hecht [a]

[a] Masonic Cancer Center and [b] Department of Medicinal Chemistry, University of Minnesota, Minneapolis, Minnesota 55455, United States.

* E-mail: lixx4803@umn.edu; Tel.: (612) 624-8187; Cancer and Cardiovascular Research Building 2-128, 2231 6th Street SE, Minneapolis, Minnesota 55455, United States.

Table of Contents:

Table S1. Levels of representative POB, PHB and methyl DNA adducts in the tissues of laboratory animals exposed to NNK or (*S*)-NNAL or (*R*)-NNAL.

Table S2: Levels of representative POB and methyl DNA adducts in the tissues of male F344 rats exposed to NNN or its enantiomers in the drinking water.

Table S1. Levels of representative POB, PHB and methyl DNA adducts in the tissues of laboratory animals exposed to NNK or (*S*)-NNAL or (*R*)-NNAL.

Animal species	Administration pathway	Exposure amount	Exposure time	Target tissue	POB, PHB, and methyl DNA base and phosphate adducts (fmol/mg DNA unless noted otherwise) formed by NNK and NNAL													Ref.
					N^6-POB-dAdo	O^2-POB-Cyt	O^6-POB-dGuo	*N*7-POB-Gua	O^2-POB-Thd	Total POB DNA phosphate adducts	N^6-PHB-dAdo	O^6-PHB-dGuo	*N*7-PHB-Gua	O^2-PHB-Thd	Total PHB DNA phosphate adducts	O^6-Me-Gua	Total methyl DNA phosphate adducts	
Female A/J mice	Single i.p. injection	10 µmol NNK in saline	8 h	Lung		0.2 ± 0.1 pmol/µmol dG	1.0 ± 0.5 pmol/µmol dG	4.2 ± 1.0 pmol/µmol dG	2.8 ± 0.5 pmol/µmol dG									[1]
			48 h			0.09 ± 0.04 pmol/µmol dG	0.7 ± 0.4 pmol/µmol dG	3.0 ± 0.3 pmol/µmol dG	2.7 ± 0.4 pmol/µmol dG									
			96 h			0.06 ± 0.04 pmol/µmol dG	0.7 ± 0.2 pmol/µmol dG	3.6 ± 1.8 pmol/µmol dG	3.0 ± 0.9 pmol/µmol dG									
Male F344 rats	Once daily s.c. injection	5.2 mg (0.025 mmol)NNK/kg body weight (low dose group)	4 consecutive days	Liver		4400 ± 781	42 ± 26	5330 ± 969	5300 ± 1088									[2]
				Lung		724 ± 54	376 ± 39	1400 ± 133	1680 ± 99									
		20.7 mg (0.1 mmol)NNK/kg body weight (high dose group)		Liver		11700 ± 2517	210 ± 38	18300 ± 2407	18400 ± 2536									
				Lung		1260 ± 253	730 ± 151	2710 ± 717	3030 ± 724									
	Dosed in the drinking water	10 ppm NNK	20 weeks	Liver		490 ± 146	ND	730 ± 225	2680 ± 643			ND	ND	454 ± 554		891 ± 379		[3-6]
				Lung		940 ± 175	20 ± 5	1060 ± 169	5070 ± 1057			15 ± 1.9	147 ± 33	322 ± 95		1910 ± 615		
				Nasal olfactory mucosa		ND	ND	63.9 ± 20.3	295 ± 22			20.4 ± 2.4	83.0 ± 26.2	357 ± 312				
				Nasal respiratory mucosa		24.7 ± 29.0	36.3 ± 32.5	433 ± 267	1617 ± 831			285 ± 1	517 ± 109	1715 ± 1155				
				Oral mucosa		ND	ND	51.3 ± 14.9	326 ± 74			ND	ND	139 ± 197				
				Pancreas		ND	ND	26.7 ± 6.7	130 ± 15			ND	13.6 ± 12.0	113 ± 10				
		10 ppm (*S*)-NNAL	20 weeks	Liver		360 ± 47	ND	470 ± 86	1910 ± 243			ND	ND	140 ± 13				
				Lung		1020 ± 73	20 ± 2	1110 ± 75	4980 ± 65			16 ± 3.8	159 ± 56	394 ± 86				
				Nasal olfactory mucosa		ND	ND	47.8 ± 3.1	235 ± 30			13.0 ± 0.3	47.5 ± 2.5	260 ± 248				
				Nasal respiratory mucosa		33.4 ± 17.6	41.1 ± 27.7	377 ± 172	1451 ± 581			272 ± 9	454 ± 85	1401 ± 913				
				Oral mucosa		ND	ND	27.8 ± 24.1	227 ± 24			ND	ND	ND				
				Pancreas		ND	ND	26.4 ± 1.2	149 ± 26			ND	ND	90.9 ± 4.6				
		10 ppm (*R*)-NNAL	20 weeks	Liver		0	ND	40 ± 5	200 ± 47			ND	757 ± 423	1570 ± 846				
				Lung		60 ± 10	0	100 ± 11	480 ± 18			455 ± 123	3770 ± 1020	8080 ± 1750				

Animal species	Administration pathway	Exposure amount	Exposure time	Target tissue	POB, PHB, and methyl DNA base and phosphate adducts (fmol/mg DNA unless noted otherwise) formed by NNK and NNAL													Ref.
					N^6-POB-dAdo	O^2-POB-Cyt	O^6-POB-dGuo	N7-POB-Gua	O^2-POB-Thd	Total POB DNA phosphate adducts	N^6-PHB-dAdo	O^6-PHB-dGuo	N7-PHB-Gua	O^2-PHB-Thd	Total PHB DNA phosphate adducts	O^6-Me-Gua	Total methyl DNA phosphate adducts	
				Nasal olfactory mucosa		ND	ND	5.4 ± 0.2	33.3 ± 4.0			23.0 ± 4.0	439 ± 81	1373 ± 880				
				Nasal respiratory mucosa		ND	ND	42.7 ± 13.5	195 ± 44			306 ± 18	3300 ± 446	9014 ± 7497				
				Oral mucosa		ND	ND	ND	3.8 ± 6.6			ND	189 ± 49	844 ± 585				
				pancreas		ND	ND	ND	11.4 ± 2.4			4.1 ± 1.1	73.0 ± 17.7	475 ± 73				
	Dosed in the drinking water	5 ppm NNK	10 weeks	Liver			ND	501 ± 53	1808 ± 840	190 ± 49		ND	335 ± 40	390 ± 185	2140 ± 131			[7-12]
				Lung			34 ± 21	970 ± 148	3591 ± 414	475 ± 95		ND	235 ± 40	968 ± 28	6390 ± 457	213 ± 27	2290 ± 546	
				Pancreas			ND	30 ± 8	102 ± 8			ND	105 ± 15	51 ± 14		ND		
			30 weeks	Liver			ND	331 ± 36	2144 ± 26	134 ± 64		ND	313 ± 79	737 ± 186	1930 ± 81			
				Lung			9 ± 9	751 ± 29	4809 ± 193	417 ± 43		ND	164 ± 44	1369 ± 94	8160 ± 654	100 ± 65	4480 ± 119	
				Pancreas			ND	27 ± 21	134 ± 28			ND	98 ± 16	89 ± 9		ND		
			50 weeks	Liver	32 ± 9		ND	257 ± 32	1531 ± 161	120 ± 36	6 ± 1	ND	295 ± 42	658 ± 107	1630 ± 166			
				Lung	99 ± 3		9 ± 2	688 ± 65	4409 ± 320	346 ± 41	35 ± 5	ND	129 ± 34	1259 ± 66	4000 ± 291	79 ± 15	3820 ± 215	
				Pancreas			ND	24 ± 5	121 ± 18			ND	-	76 ± 38		ND		
			70 weeks	Liver			ND	230 ± 90	910 ± 6	89 ± 4		ND	194 ± 32	272 ± 86	878 ± 210			
				Lung			4 ± 2	315 ± 75	1983 ± 467	218 ± 15		ND	62 ± 15	807 ± 142	3950 ± 223	34	4510 ± 129	
				Pancreas			ND	19 ± 3	109 ± 17			ND	90	54 ± 5		ND		
		5 ppm (*S*)-NNAL	10 weeks	Liver														
				Lung			9 ± 5	609 ± 143	2564 ± 648	4650 ± 356		ND	138 ± 17	709 ± 29	3670 ± 67	269 ± 178	1120 ± 217	
				Pancreas			ND	23 ± 9	80 ± 8			ND	-	43 ± 1		ND		
			30 weeks	Liver														
				Lung			10 ± 3	624 ± 66	3319 ± 365	2910 ± 109		ND	125 ± 14	1083 ± 27	4180 ± 119	114 ± 25	1020 ± 290	
				Pancreas			ND	17 ± 4	102 ± 11			ND	74 ± 68	75 ± 12		ND		
			50 weeks	Liver	27 ± 6						5 ± 1							
				Lung	130 ± 1		10 ± 5	568 ± 68	3951 ± 328	1310 ± 77	36 ± 8	ND	100 ± 25	1152 ± 41	3480 ± 79	78 ± 29	872 ± 309	
				Pancreas			ND	22 ± 14	98 ± 14			ND	73	64 ± 22		ND		
			70 weeks	Liver														
				Lung			4 ± 2	512 ± 70	3949 ± 256	1180 ± 204		ND	116 ± 28	1155 ± 310	3820 ± 582	49 ± 22	995 ± 258	
				Pancreas			ND	25 ± 5	101 ± 18			ND	105	51 ± 13		ND		
		5 ppm (*R*)-NNAL	10 weeks	Liver														

Animal species	Administration pathway	Exposure amount	Exposure time	Target tissue	POB, PHB, and methyl DNA base and phosphate adducts (fmol/mg DNA unless noted otherwise) formed by NNK and NNAL													Ref.
					N^6-POB-dAdo	O^2-POB-Cyt	O^6-POB-dGuo	$N7$-POB-Gua	O^2-POB-Thd	Total POB DNA phosphate adducts	N^6-PHB-dAdo	O^6-PHB-dGuo	$N7$-PHB-Gua	O^2-PHB-Thd	Total PHB DNA phosphate adducts	O^6-Me-Gua	Total methyl DNA phosphate adducts	
				Lung			ND	62 ± 19	261 ± 31	136 ± 32		ND	869 ± 143	4009 ± 707	4530 ± 368	5 ± 2	874 ± 146	
				Pancreas			ND	4	8 ± 2			ND	159 ± 17	181 ± 50		ND		
				Liver														
			30 weeks	Lung			ND	75 ± 24	415 ± 77	135 ± 30		ND	861 ± 161	6581 ± 481	6670 ± 211	17	1430 ± 140	
				Pancreas			ND	3	10 ± 1			ND	172 ± 32	431 ± 37		ND		
				Liver	4 ± 1						24 ± 2							
			50 weeks	Lung	16 ± 7		ND	136 ± 10	584 ± 45	175 ± 17	125 ± 34	ND	379 ± 19	6291 ± 885	6280 ± 336	7	1100 ± 257	
				Pancreas			ND	3	13 ± 1			ND	124 ± 1	369 ± 80		ND		
				Liver														
			70 weeks	Lung			ND	45 ± 16	500 ± 111	46		ND	378 ± 105	5792 ± 1763	6920 ± 436	4	763 ± 90	
				Pancreas			ND	2	18 ± 2			ND	138 ± 36	436 ± 99		ND		

Table S2. Levels of representative POB and methyl DNA adducts in the tissues of male F344 rats exposed to NNN or its enantiomers in the drinking water.

Exposure amount	Exposure time	Target tissue	DNA base and phosphate adducts (fmol/mg DNA) formed by NNN or its enantiomers							Ref.
			*O*²-POB-Cyt	*O*⁶-POB-dGuo	*N7*-POB-Gua	*O*²-POB-Thd	Total POB DNA phosphate adducts	Py-Py-dI	*O*⁶-HPB-dAdo	
10 ppm (*S*)-NNN	20 weeks	Liver	40 ± 13	-	70 ± 8	300 ± 32				[13, 14]
		Lung	30 ± 6	-	50 ± 6	230 ± 24				
		Esophagus	230 ± 39	-	360 ± 71	420 ± 4				
		Nasal olfactory mucosa	ND	ND	423 ± 623	1990 ± 2860				
		Nasal respiratory mucosa	14.4 ± 24.9	ND	591 ± 95	2270 ± 130				
		Oral mucosa	ND	ND	198 ± 159	277 ± 225				
10 ppm (*R*)-NNN	20 weeks	Liver	0	-	0	40 ± 4				
		Lung	80 ± 21	-	100 ± 11	470 ± 20				
		Esophagus	30 ± 8	-	140 ± 41	90 ± 12				
		Nasal olfactory mucosa	9.0 ± 7.8	ND	200 ± 48	1200 ± 240				
		Nasal respiratory mucosa	53.2 ± 48.4	4.1 ± 7.1	996 ± 292	4620 ± 1170				
		Oral mucosa	ND	ND	49.4 ± 42.8	89.7 ± 11.5				
14 ppm (*S*)-NNN	10 weeks	Liver	-	-	225 ± 22	503 ± 80	340 ± 59			[15, 16]
		Lung	-	-	97 ± 3	230 ± 7	-			
		Esophagus	-	< LOD	1270 ± 310	756 ± 125	-			
		Nasal olfactory mucosa	-	-	100 ± 4	331 ± 1	150 ± 48			
		Nasal respiratory mucosa	-	< LOD	1303 ± 25	5419 ± 358	1740 ± 206			
		Oral mucosa	ND	ND	271 ± 27	321 ± 49	260 ± 60			
	30 weeks	Liver	-	-	156 ± 12	533 ± 60	410 ± 84			
		Lung	-	-	85 ± 6	265 ± 11	-			
		Esophagus	-	< LOD	1357 ± 135	1083 ± 64	3910 ± 715			
		Nasal olfactory mucosa	-	-	114 ± 1	474 ± 27	170 ± 41			
		Nasal respiratory mucosa	-	< LOD	1124 ± 6	5676 ± 654	1720 ± 156			
		Oral mucosa	ND	ND	341 ± 31	408 ± 17	200 ± 30			
	50 weeks	Liver	-	-	90 ± 13	374 ± 46	190 ± 38			
		Lung	-	-	64 ± 6	224 ± 13	-			
		Esophagus	-	< LOD	1384 ± 226	1166 ± 231	5400 ± 317			
		Nasal olfactory mucosa	-	-	125 ± 3	475 ± 19	150 ± 7			
		Nasal respiratory mucosa	-	< LOD	1019 ± 107	5839 ± 709	2160 ± 495			

Exposure amount	Exposure time	Target tissue	DNA base and phosphate adducts (fmol/mg DNA) formed by NNN or its enantiomers							Ref.
			O^2-POB-Cyt	O^6-POB-dGuo	*N*7-POB-Gua	O^2-POB-Thd	Total POB DNA phosphate adducts	Py-Py-dI	O^6-HPB-dAdo	
		Oral mucosa	ND	ND	393 ± 37	458 ± 37	120 ± 27			
	70 weeks	Liver	-	-	148 ± 50	478 ± 85	200 ± 50			
		Lung	-	-	63 ± 8	198 ± 9	-			
		Esophagus	-	< LOD	1965 ± 610	1689 ± 450	4970			
		Nasal olfactory mucosa	-	-	162 ± 5	553 ± 74	250 ± 87			
		Nasal respiratory mucosa	-	< LOD	1142 ± 189	6941 ± 1299	1910 ± 198			
		Oral mucosa	ND	ND	387 ± 74	534 ± 162	290 ± 156			
14 ppm (*R*)-NNN	10 weeks	Liver	-	-	34 ± 8	86 ± 9	< LOD			
		Lung	-	-	191 ± 11	514 ± 21	-			
		Esophagus	-	-	513 ± 54	223 ± 3	1400 ± 229			
		Nasal olfactory mucosa	-	-	332 ± 3	1367 ± 130	340 ± 74			
		Nasal respiratory mucosa	-	< LOD	745 ± 66	2504 ± 177	2830 ± 355			
		Oral mucosa	-	-	108 ± 0	76 ± 4	40 ± 10			
	30 weeks	Liver	-	-	36 ± 25	77 ± 7	< LOD			
		Lung	-	-	167 ± 18	591 ± 79	-			
		Esophagus	-	-	515 ± 119	206 ± 1	2110 ± 1270			
		Nasal olfactory mucosa	-	-	318 ± 16	1567 ± 43	670 ± 121			
		Nasal respiratory mucosa	-	< LOD	805 ± 95	3259 ± 222	3550 ± 720			
		Oral mucosa	-	-	124 ± 3	88 ± 5	60 ± 54			
	50 weeks	Liver	-	-	17 ± 3	69 ± 10	< LOD			
		Lung	-	-	144 ± 7	574 ± 49	-			
		Esophagus	-	-	772 ± 208	176 ± 25	970 ± 211			
		Nasal olfactory mucosa	-	-	314 ± 7	1532 ± 58	520 ± 121			
		Nasal respiratory mucosa	-	< LOD	719 ± 100	3000 ± 706	2100 ± 327			
		Oral mucosa	-	-	118 ± 8	90 ± 7	30 ± 15			
	70 weeks	Liver	-	-	18 ± 2	64 ± 11	< LOD			
		Lung	-	-	114 ± 4	454 ± 36	-			
		Esophagus	-	-	481 ± 152	221 ± 51	990 ± 246			
		Nasal olfactory mucosa	-	-	359 ± 63	1804 ± 316	520 ± 151			
		Nasal respiratory mucosa	-	< LOD	763 ± 137	2955 ± 686	3680 ± 934			
		Oral mucosa	-	-	170 ± 6	159 ± 2	70 ± 48			
50 ppm (*S*)-NNN	3 weeks	Liver	ND	ND	26 ± 4	110 ± 5				[17]

Exposure amount	Exposure time	Target tissue	DNA base and phosphate adducts (fmol/mg DNA) formed by NNN or its enantiomers							Ref.
			*O*²-POB-Cyt	*O*⁶-POB-dGuo	*N*7-POB-Gua	*O*²-POB-Thd	Total POB DNA phosphate adducts	Py-Py-dI	*O*⁶-HPB-dAdo	
		Lung	4.7 ± 0.3	ND	46 ± 8	118 ± 9				
		Esophagus	74	4.5 ± 1.7	309 ± 31	287 ± 32				
		Nasal olfactory mucosa	4 ± 1	ND	52 ± 10	140 ± 14				
		Nasal respiratory mucosa	22 ± 3	ND	283 ± 39	759 ± 34				
		Oral mucosa	16	2.2 ± 0.7	106 ± 28	138 ± 29				
100 ppm (*S*)-NNN	3 weeks	Liver	ND	ND	57 ± 6	239 ± 35				
		Lung	6 ± 1	ND	84 ± 4	208 ± 14				
		Esophagus	57	8.5 ± 1.3	487 ± 71	474 ± 82				
		Nasal olfactory mucosa	8 ± 1	ND	97 ± 13	264 ± 3				
		Nasal respiratory mucosa	51 ± 12	9 ± 1	678 ± 186	1710 ± 330				
		Oral mucosa	21	3.3 ± 0.6	162 ± 39	200 ± 47				
500 ppm (*S*)-NNN	3 weeks	Liver	9	ND	108 ± 14	437 ± 26				
		Lung	8 ± 1	ND	141 ± 17	332 ± 32				
		Esophagus	135 ± 27	16.7 ± 4.9	1110 ± 160	1050 ± 110				
		Nasal olfactory mucosa	14 ± 6	3	165 ± 44	453 ± 85				
		Nasal respiratory mucosa	87 ± 18	23 ± 4	1230 ± 360	3110 ± 520				
		Oral mucosa	32	5.1 ± 0.8	257 ± 28	307 ± 34				
50 ppm (*R*)-NNN	3 weeks	Liver	ND	ND	4 ± 1	15 ± 2				
		Lung	6 ± 1	ND	107 ± 29	302 ± 49				
		Esophagus	ND	2.4 ± 1.0	129 ± 16	66 ± 1				
		Nasal olfactory mucosa	14 ± 3	ND	135 ± 41	443 ± 55				
		Nasal respiratory mucosa	38 ± 6	8	489 ± 172	1510 ± 270				
		Oral mucosa	ND	0.8 ± 0.3	39 ± 13	24 ± 3				
100 ppm (*R*)-NNN	3 weeks	Liver	ND	ND	10 ± 1	34 ± 5				
		Lung	14 ± 3	ND	182 ± 11	563 ± 69				
		Esophagus	ND	5.5 ± 1.2	212 ± 35	119 ± 18				
		Nasal olfactory mucosa	31 ± 12	4 ± 1	325 ± 135	1110 ± 310				
		Nasal respiratory mucosa	95 ± 26	22 ± 3	1090 ± 430	3440 ± 860				
		Oral mucosa	7	2.0 ± 0.6	69 ± 15	50 ± 11				
500 ppm (*R*)-NNN	3 weeks	Liver	ND	ND	21 ± 14	64 ± 12				
		Lung	29 ± 5	ND	366 ± 26	1130 ± 80				
		Esophagus	48	14.7 ± 3.3	400 ± 40	256 ± 43				

Exposure amount	Exposure time	Target tissue	DNA base and phosphate adducts (fmol/mg DNA) formed by NNN or its enantiomers							Ref.
			*O*²-POB-Cyt	*O*⁶-POB-dGuo	*N*7-POB-Gua	*O*²-POB-Thd	Total POB DNA phosphate adducts	Py-Py-dI	*O*⁶-HPB-dAdo	
		Nasal olfactory mucosa	51 ± 18	9 ± 1	615 ± 158	2150 ± 210				
		Nasal respiratory mucosa	192 ± 41	31 ± 5	2090 ± 790	6410 ± 1540				
		Oral mucosa	10	3.9 ± 1.0	104 ± 18	104 ± 28				
50 ppm racemic NNN	3 weeks	Liver	ND	ND	30 ± 2	132 ± 6		56 ± 14 fmol/µmol dGuo	74 ± 4 fmol/µmol dGuo	[17-19]
		Lung	11 ± 2	ND	147 ± 42	420 ± 74		580 ± 41	110 ± 18	
		Esophagus	62	11.2 ± 1.6	417 ± 65	354 ± 23		129 ± 13		
		Nasal olfactory mucosa	15 ± 3	3	178 ± 33	590 ± 78		458 ± 37		
		Nasal respiratory mucosa	71 ± 9	16 ± 3	873 ± 103	2500 ± 180		869 ± 14		
		Oral mucosa	14	3.5 ± 1.0	116 ± 31	134 ± 23		31 ± 3.8		
100 ppm racemic NNN	3 weeks	Liver	ND	ND	74 ± 20	268 ± 14		157 ± 42	84 ± 20	
		Lung	18 ± 4	ND	269 ± 36	748 ± 90		1045 ± 69	176 ± 34	
		Esophagus	90 ± 7	18.4 ± 3.3	679 ± 61	593 ± 98		158 ± 26		
		Nasal olfactory mucosa	35 ± 13	6 ± 2	404 ± 155	1250 ± 330		957 ± 7.8		
		Nasal respiratory mucosa	144 ± 14	24 ± 6	1660 ± 300	5090 ± 500		1451 ± 260		
		Oral mucosa	24 ± 6	5.2 ± 0.1	195 ± 22	225 ± 41		60 ± 1.6		
500 ppm racemic NNN	3 weeks	Liver	12	ND	111 ± 19	472 ± 53		412 ± 53	233 ± 84	
		Lung	21 ± 4	ND	335 ± 28	985 ± 113		2018 ± 200	328 ± 44	
		Esophagus	172 ± 51	26.4 ± 10.8	1300 ± 150	1180 ± 60		232 ± 89		
		Nasal olfactory mucosa	68 ± 21	12 ± 4	794 ± 204	2420 ± 750		2458 ± 190		
		Nasal respiratory mucosa	292 ± 43	41 ± 3	3240 ± 500	9420 ± 1300		2015 ± 80		
		Oral mucosa	33 ± 3	8.6 ± 1.5	269 ± 41	295 ± 64		100 ± 7.1		

References

1. Urban, A.M.; Upadhyaya, P.; Cao, Q.; Peterson, L.A., Formation and repair of pyridyloxobutyl DNA adducts and their relationship to tumor yield in A/J mice. *Chem. Res. Toxicol.* **2012,** 25, (10), 2167-78.
2. Lao, Y.; Villalta, P.W.; Sturla, S.J.; Wang, M.; Hecht, S.S., Quantitation of pyridyloxobutyl DNA adducts of tobacco-specific nitrosamines in rat tissue DNA by high-performance liquid chromatography-electrospray ionization-tandem mass spectrometry. *Chem. Res. Toxicol.* **2006,** 19, (5), 674-82.
3. Lao, Y.; Yu, N.; Kassie, F.; Villalta, P.W.; Hecht, S.S., Formation and accumulation of pyridyloxobutyl DNA adducts in F344 rats chronically treated with 4-(methylnitrosamino)-1-(3-pyridyl)-1-butanone and enantiomers of its metabolite, 4-(methylnitrosamino)-1-(3-pyridyl)-1-butanol. *Chem. Res. Toxicol.* **2007,** 20, (2), 235-45.
4. Zhang, S.; Wang, M.; Villalta, P.W.; Lindgren, B.R.; Upadhyaya, P.; Lao, Y.; Hecht, S.S., Analysis of pyridyloxobutyl and pyridylhydroxybutyl DNA adducts in extrahepatic tissues of F344 rats treated chronically with 4-(methylnitrosamino)-1-(3-pyridyl)-1-butanone and enantiomers of 4-(methylnitrosamino)-1-(3-pyridyl)-1-butanol. *Chem. Res. Toxicol.* **2009,** 22, (5), 926-36.
5. Upadhyaya, P.; Kalscheuer, S.; Hochalter, J.B.; Villalta, P.W.; Hecht, S.S., Quantitation of pyridylhydroxybutyl-DNA adducts in liver and lung of F-344 rats treated with 4-(methylnitrosamino)-1-(3-pyridyl)-1-butanone and enantiomers of its metabolite 4-(methylnitrosamino)-1-(3-pyridyl)-1-butanol. *Chem. Res. Toxicol.* **2008,** 21, (7), 1468-76.
6. Upadhyaya, P.; Lindgren, B.R.; Hecht, S.S., Comparative levels of O^6-methylguanine, pyridyloxobutyl-, and pyridylhydroxybutyl-DNA adducts in lung and liver of rats treated chronically with the tobacco-specific carcinogen 4-(methylnitrosamino)-1-(3-pyridyl)-1-butanone. *Drug Metab. Dispos.* **2009,** 37, (6), 1147-51.
7. Carlson, E.S.; Upadhyaya, P.; Villalta, P.W.; Ma, B.; Hecht, S.S., Analysis and identification of 2'-deoxyadenosine-derived adducts in lung and liver DNA of F-344 rats treated with the tobacco-specific carcinogen 4-(methylnitrosamino)-1-(3-pyridyl)-1-butanone and enantiomers of its metabolite 4-(methylnitrosamino)-1-(3-pyridyl)-1-butanol. *Chem. Res. Toxicol.* **2018,** 31, (5), 358-370.
8. Ma, B.; Villalta, P.W.; Zarth, A.T.; Kotandeniya, D.; Upadhyaya, P.; Stepanov, I.; Hecht, S.S., Comprehensive high-resolution mass spectrometric analysis of DNA phosphate adducts formed by the tobacco-specific lung carcinogen 4-(methylnitrosamino)-1-(3-pyridyl)-1-butanone. *Chem. Res. Toxicol.* **2015,** 28, (11), 2151-9.
9. Ma, B.; Zarth, A.T.; Carlson, E.S.; Villalta, P.W.; Upadhyaya, P.; Stepanov, I.; Hecht, S.S., Identification of more than 100 structurally unique DNA-phosphate adducts formed during rat lung carcinogenesis by the tobacco-specific nitrosamine 4-(methylnitrosamino)-1-(3-pyridyl)-1-butanone. *Carcinogenesis* **2018,** 39, (2), 232-241.
10. Balbo, S.; Johnson, C.S.; Kovi, R.C.; James-Yi, S.A.; O'Sullivan, M.G.; Wang, M.; Le, C.T.; Khariwala, S.S.; Upadhyaya, P.; Hecht, S.S., Carcinogenicity and DNA adduct formation of 4-(methylnitrosamino)-1-(3-pyridyl)-1-butanone and enantiomers of its metabolite 4-(methylnitrosamino)-1-(3-pyridyl)-1-butanol in F-344 rats. *Carcinogenesis* **2014,** 35, (12), 2798-806.
11. Ma, B.; Zarth, A.T.; Carlson, E.S.; Villalta, P.W.; Stepanov, I.; Hecht, S.S., Pyridylhydroxybutyl and pyridyloxobutyl DNA phosphate adduct formation in rats treated chronically with enantiomers of the tobacco-specific nitrosamine metabolite 4-(methylnitrosamino)-1-(3-pyridyl)-1-butanol. *Mutagenesis* **2017,** 32, (6), 561-570.
12. Ma, B.; Zarth, A.T.; Carlson, E.S.; Villalta, P.W.; Upadhyaya, P.; Stepanov, I.; Hecht, S.S., Methyl DNA phosphate adduct formation in rats treated chronically with 4-(methylnitrosamino)-1-(3-pyridyl)-1-butanone and enantiomers of its metabolite 4-(methylnitrosamino)-1-(3-pyridyl)-1-butanol. *Chem. Res. Toxicol.* **2018,** 31, (1), 48-57.
13. Lao, Y.; Yu, N.; Kassie, F.; Villalta, P.W.; Hecht, S.S., Analysis of pyridyloxobutyl DNA adducts in F344 rats chronically treated with (*R*)- and (*S*)-*N'*-nitrosonornicotine. *Chem. Res. Toxicol.* **2007,** 20, (2), 246-56.
14. Zhang, S.; Wang, M.; Villalta, P.W.; Lindgren, B.R.; Lao, Y.; Hecht, S.S., Quantitation of pyridyloxobutyl DNA adducts in nasal and oral mucosa of rats treated chronically with enantiomers of *N'*-nitrosonornicotine. *Chem. Res. Toxicol.* **2009,** 22, (5), 949-56.

15. Zhao, L.; Balbo, S.; Wang, M.; Upadhyaya, P.; Khariwala, S.S.; Villalta, P.W.; Hecht, S.S., Quantitation of pyridyloxobutyl-DNA adducts in tissues of rats treated chronically with (*R*)- or (*S*)-*N'*-nitrosonornicotine (NNN) in a carcinogenicity study. *Chem. Res. Toxicol.* **2013,** 26, (10), 1526-35.
16. Li, Y.; Ma, B.; Cao, Q.; Balbo, S.; Zhao, L.; Upadhyaya, P.; Hecht, S.S., Mass spectrometric quantitation of pyridyloxobutyl DNA phosphate adducts in rats chronically treated with *N'*-nitrosonornicotine. *Chem. Res. Toxicol.* **2019,** 32, (4), 773-783.
17. Yang, J.; Villalta, P.W.; Upadhyaya, P.; Hecht, S.S., Analysis of O^6-[4-(3-pyridyl)-4-oxobut-1-yl]-2'-deoxyguanosine and other DNA adducts in rats treated with enantiomeric or racemic *N'*-ntrosonornicotine. *Chem. Res. Toxicol.* **2016,** 29, (1), 87-95.
18. Zarth, A.T.; Upadhyaya, P.; Yang, J.; Hecht, S.S., DNA adduct formation from metabolic 5'-hydroxylation of the tobacco-specific carcinogen *N'*-nitrosonornicotine in human enzyme systems and in rats. *Chem. Res. Toxicol.* **2016,** 29, (3), 380-9.
19. Li, Y.; Hecht, S.S., Identification of an *N'*-nitrosonornicotine-specific deoxyadenosine adduct in rat liver and lung DNA. *Chem. Res. Toxicol.* **2021,** 34, (4), 992-1003.
